# Mosloflavone-Resveratrol Hybrid TMS-HDMF-5z Exhibits Potent *In Vitro* and *In Vivo* Anti-Inflammatory Effects Through NF-κB, AP-1, and JAK/STAT Inactivation

**DOI:** 10.3389/fphar.2022.857789

**Published:** 2022-04-21

**Authors:** Seo-Yeon Kim, Ahmed H.E. Hassan, Kyung-Sook Chung, Su-Yeon Kim, Hee-Soo Han, Hwi-Ho Lee, Seang-Hwan Jung, Kwang-Young Lee, Ji-Sun Shin, Eungyeong Jang, Seolmin Yoon, Yong Sup Lee, Kyung-Tae Lee

**Affiliations:** ^1^ Department of Pharmaceutical Biochemistry, College of Pharmacy, Kyung Hee University, Seoul, South Korea; ^2^ Department of Life and Nanopharmaceutical Sciences, Graduate School, Kyung Hee University, Seoul, South Korea; ^3^ Medicinal Chemistry Laboratory, College of Pharmacy, Kyung Hee University, Seoul, South Korea; ^4^ Department of Medicinal Chemistry, Faculty of Pharmacy, Mansoura University, Mansoura, Egypt; ^5^ Department of Internal Medicine, College of Korean Medicine, Kyung Hee University, Seoul, South Korea; ^6^ Department of Internal Medicine, Kyung Hee University Korean Medicine Hospital, Seoul, South Korea

**Keywords:** TMS-HDMF-5z, macrophages, carrageenan-induced edema, AP-1, NF-κB

## Abstract

TMS-HDMF-5z is a hybrid of the natural products mosloflavone and resveratrol. It was discovered to show potent inhibitory effects against lipopolysaccharide (LPS)-induced production of inflammatory mediators in RAW 264.7 macrophages. However, its mechanism of action is unknown. Hence this study aimed to demonstrate and explore *in vitro* and *in vivo* anti-inflammatory effects of TMS-HDMF-5z and its mechanism of action employing RAW 264.7 macrophages and carrageenan-induced hind paw edema. This work revealed that TMS-HDMF-5z suppressed the LPS-induced inducible nitric-oxide synthase (iNOS) and cyclooxygenase-2 (COX-2) at the protein, mRNA, and promoter binding levels and tumor necrosis factor-α (TNF-α), interleukin (IL)-1β, and IL-6, and interferon-β (IFN-β) at the mRNA expression in RAW 264.7 macrophages. The results showed that TMS-HDMF-5z reduced the transcription and DNA binding activities of nuclear factor-κB (NF-κB) through inhibiting nuclear translocation of p65 and phosphorylation of κB inhibitor α (IκBα), IκB kinase (IKK), and TGF-β activated kinase 1 (TAK1). Additionally, TMS-HDMF-5z attenuated the LPS-induced transcriptional and DNA binding activities of activator protein-1 (AP-1) by suppressing nuclear translocation of phosphorylated c-Fos, c-Jun, and activating transcription factor 2 (ATF2). TMS-HDMF-5z also reduced the LPS-induced phosphorylation of Janus kinase 1/2 (JAK1/2), signal transducers and activators of transcription 1/3 (STAT1/3), p38 mitogen-activated protein kinase (MAPK), and MAPK-activated protein kinase 2 (MK2). In rats, TMS-HDMF-5z alleviated carrageenan-induced hind paw edema through the suppressing iNOS and COX-2 *via* NF-κB, AP-1, and STAT1/3 inactivation. Collectively, the TMS-HDMF-5z-mediated inhibition of NF-κB, AP-1, and STAT1/3 offer an opportunity for the development of a potential treatment for inflammatory diseases.

## Graphical Abstract

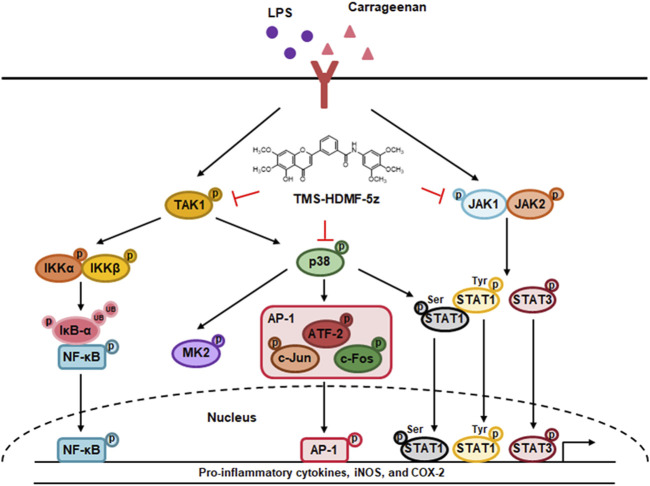

## Introduction

Multiple signaling pathways contribute to inflammatory response through modulation of inflammatory processes with pro- and anti-inflammatory mediators in macrophages and neutrophils (Lawrence, 2009). Within the core, macrophages are indispensable for host defense, acting directly as effector cells of the innate immune system or indirectly by engaging elements of the adaptive immune systems (Twum et al., 2017). Toll-like receptors (TLRs) are distinctly required for pathogen recognition by the innate immune system and mediate the production of inflammatory factors and also regulate immune responses. In response to lipopolysaccharide (LPS) treatment, which targets the TLR4, macrophages produce pro-inflammatory mediators such as nitric oxide (NO), prostaglandin E_2_ (PGE_2_), and pro-inflammatory cytokines including tumor necrosis factor-α (TNF-α), interleukin (IL)-1β, IL-6, and interferon-β (IFN-β). The dysregulation of inflammatory processes plays a major role in the pathogenesis of various diseases including cancer, endothelial injury, and oxidative stress (Szarka et al., 2010).

NF-κB plays a critical role in regulating the activation, survival, and differentiation of innate immune cells and inflammatory T cells. NF-κB forms a p65/p50 heterodimer and is activated by TGF-β activated kinase 1 (TAK1), regulating the inhibition of IκB kinase (IKK) complex and mitogen-activated protein kinase (MAPK) signaling cascades (Lawrence, 2009). After the inflammatory stimulus, the inhibitor of κB (IκB) is separated from the rest of the IKK complex and then IκBs are ubiquitinated by E3 ubiquitin ligase and degraded by the proteasome, causing nuclear translocation of NF-κB p65/p50 heterodimers. The activator protein-1 (AP-1) signaling pathway, a complex of c-Fos and c-Jun proteins, is triggered by inflammatory signaling and mainly activated by the MAPK signaling proteins such as p38 MAPK, c-Jun NH2-terminal kinase (JNK), and extracellular signal-regulated kinase (ERK), which promote the expression of genes encoding pro-inflammatory cytokines (Cargnello and Roux, 2011). The Janus kinase-signal transducer and activator of the transcription (JAK-STAT) pathway are modulated by exposure to inflammatory molecules, such as various cytokines and LPS, and are important for regulating the immune system (O'Shea and Plenge, 2012). In response to diverse stimuli, JAKs phosphorylate specific tyrosine and thus trigger the activation of STAT monomers, which leads to nuclear translocation and DNA binding by STAT dimers (O'Shea and Plenge, 2012).

Mosloflavone (5-hydroxy-6,7-dimethoxyflavone; HDMF) and the structurally-related TMF (5,6,7-trimethoxyflavone) are two natural products isolated from several plants and were found to possess anti-inflammatory properties (Singh et al., 2013). TMF is a key intermediate in the synthesis of baicalein and oroxylin A derivatives (Singh et al., 2013) that elicit various pharmacological activities including antiviral (Hayashi et al., 1997), anticancer (Liao and Hu, 2004), and antibacterial (Suresh Babu et al., 2005) activities. In addition, TMF and its derivatives were found to have anti-inflammatory potential (Kim et al., 2012). Meanwhile, resveratrol, another naturally occurring plant polyphenol found in berries and red wines, was reported to show a plethora of biological activities including anti-inflammatory and anti-microbial effects (Novelle et al., 2015). Literature has reported that the protective effects of resveratrol are mediated through multiple targets including STAT3, MAPK, and peroxisome proliferator-activated receptor-γ (PPAR-γ) pathways. Nevertheless, it is now established that resveratrol is not effective or is restricted in clinical research because of low bioavailability and rapid plasma clearance despite its reported extensive studies. To circumvent these hurdles, the anti-inflammatory properties of hybrids of resveratrol amide analogs with mosloflavone or TMF were preliminary explored in LPS-induced pro-inflammatory mediators. Among them, TMS-HDMF-5z (3-(5-hydroxy-6,7-dimethoxy-4-oxo-4*H*-chromen-2-yl)-*N*-(3,4,5-trimethoxy phenyl)benzamide) was the most potential significantly decreasing NO, PGE_2_, and pro-inflammatory cytokine production and p38 MAPK phosphorylation (Hassan et al., 2019). Nevertheless, the mechanism underlying suppression of these pro-inflammatory mediators is unknown. This study embarks on *in vitro* and *in vivo* investigation of the anti-inflammatory activities and involved molecular mechanisms of TMS-HDMF-5z in LPS-stimulated RAW 264.7 macrophages and a carrageenan-induced paw edema in rats.

## Materials and Methods

### Materials and Cell Culture

**FIGURE 1:**
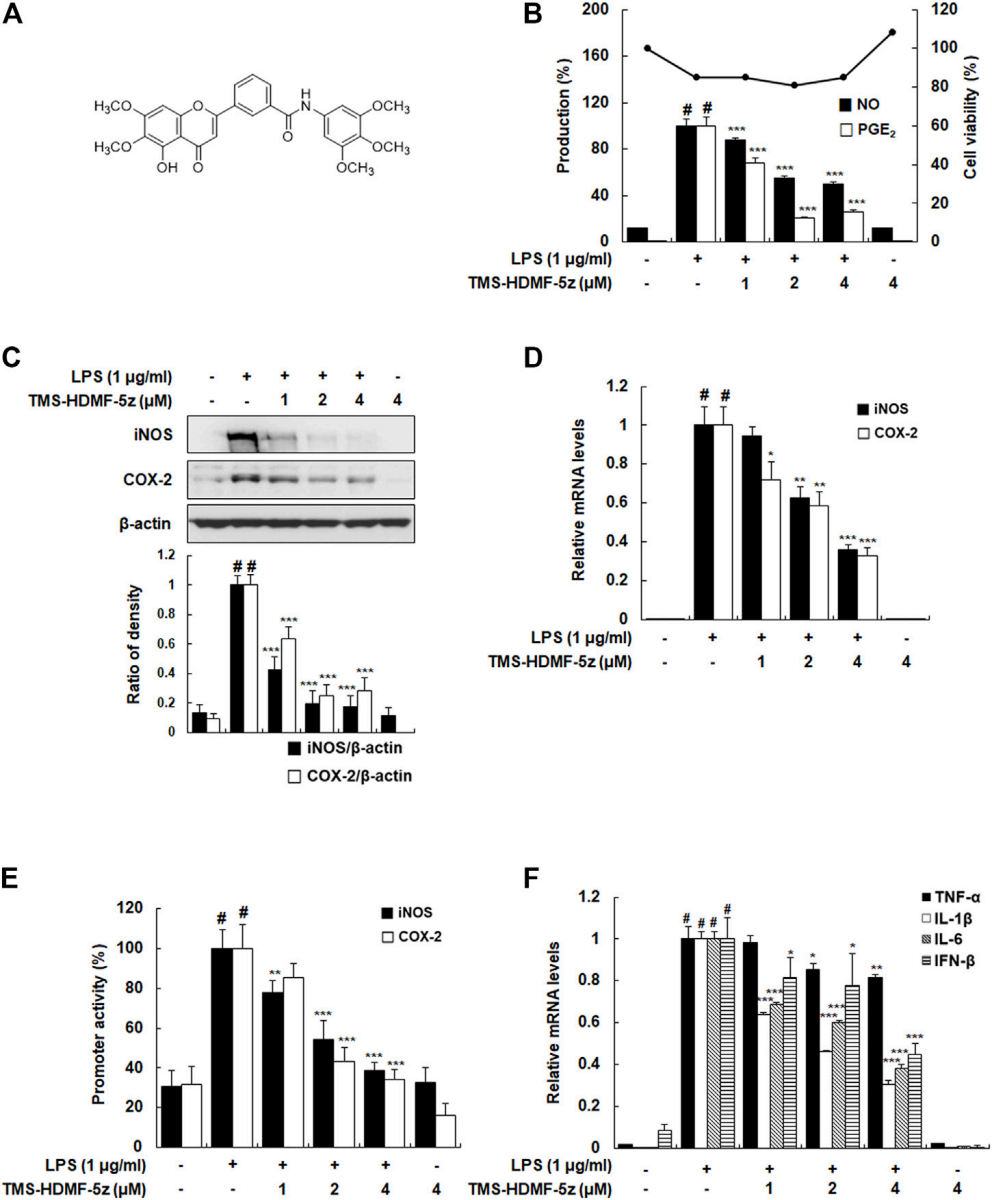
Inhibitory effects of TMS-HDMF-5z on LPS-induced production of NO and PGE_2_, expression and promoter activities of iNOS and COX-2, and mRNA expression of TNF-α, IL-1β, IL-6, and IFN-β in RAW 264.7 macrophages. **(A)** Chemical structure of TMS-HDMF-5z. **(B)** NO and PGE_2_ production was determined using the Griess reaction and an EIA kit (Enzo Life Sciences, Inc., NY, United States), respectively. Cell viability was analyzed by MTT assay. **(C)** The protein expression of iNOS and COX-2 was detected by western blotting. The relative optical density ratio was determined using a densitometric analysis program (Bio-Rad Quantity One^®^ Software, version 4.6.3 (Basic), Bio-Rad Laboratories Inc., CA, United States), normalized to the internal control. **(D)** The mRNA levels of iNOS and COX-2 were quantified by qRT-PCR. The levels of iNOS and COX-2 were adjusted by *ß*-actin expression. **(E)** The promoter activities of iNOS and COX-2 were determined using luciferase assay systems. **(F)** The mRNA levels of TNF-α, IL-1β, IL-6, and IFN-β were quantified by qRT-PCR. The expression levels of TNF-α, IL-1β, IL-6, and IFN-β were adjusted by *ß*-actin expression. The values shown are the mean ± S.D. of three independent experiments. *p* < 0.05 *vs*. the control group, *p < 0.05, **p < 0.01, ***p < 0.001 *vs*. LPS-treated cells.

TMS-HDMF-5z (Figure 1A; HPLC purity> 98%) was obtained as previously reported (Hassan et al., 2019). Dulbecco’s modified Eagle medium (DMEM) medium, fetal bovine serum (FBS), penicillin, and streptomycin were obtained from Life Technologies (NY, United States). Dithiothreitol (DTT), phenylmethylsulfonyl fluoride (PMSF), sodium fluoride (NaF), LPS (*Escherichia coli*, serotype 0111:B4), carrageenan lambda, celecoxib, croton oil, 3-(4,5-Dimethylthizol-2-yl)-2,5- diphenyltetrazolium bromide (MTT), L-N^6^-(1-iminoethyl)lysine (L-NIL), *N*-[2-(cyclohexyloxy)-4-nitrophenyl]-methanesulfonamide (NS398), sodium bicarbonate, HEPES, kolliphor EL, and all other reagents were obtained from Sigma-Aldrich (St. Louis, MO, United States). The COX-2 antibody was purchased from BD Pharmingen (San Diego, CA, United States). Antibodies of iNOS, p65, IKKβ, PARP-1, α-tubulin, IκBα, p38 MAPK, p-JNK, JNK, p-ERK, ERK, p-JAK1, p-JAK2, c-Fos, p-c-Jun, c-Jun, p-MK2, MK2, and *ß*-actin were obtained from Santa Cruz Biotechnology (Santa Cruz, CA, United States). Antibodies of p-IKKα/β, p-IκBα, p-p38 MAPK, JAK1, JAK2, p-c-Fos, p-p65, p-TAK1, and TAK1 were obtained from Cell Signaling Technologies (Danvers, MA, United States). The horse-radish peroxidase (HRP)-conjugated anti-mouse and anti-rabbit antibodies were obtained from Jackson Laboratory (MN, United States). The primers of iNOS, COX-2, TNF-α, IL-1β, IL-6, IFN-β, and *ß*-actin were obtained from Bioneer (Seoul, Republic of Korea). All chemicals and reagents above were diluted to working concentrations prior to use.

RAW 264.7 murine macrophages cell line was obtained from the Korea Cell Line Bank (Seoul, Republic of Korea). RAW 264.7 cells were incubated in DMEM containing 10% FBS and 1% penicillin-streptomycin at 37°C with 5% CO_2_. Cells were pretreated with TMS-HDMF-5z (1, 2, or 4 μM) before 1 h and were stimulated with LPS (1 μg/ml) at various times. TMS-HDMF-5z was dissolved in DMSO (Sigma Chemical Co., D2438) up to its total concentration of below 0.05%. Here, DMSO (0.05%) served for vehicle control with no cytotoxic effects on RAW 264.7 cells. Cell lines were routinely checked for *mycoplasma* contamination by e-Myco™ plus *Mycoplasma* PCR Detection Kit (Intron Biotechnology, Seongnam, Republic of Korea).

### NO and PGE_2_ Production and Cell Viability Assay

RAW 264.7 macrophages were seeded at 2 × 10^5^/ml/well in a 24-well plate and incubated with TMS-HDMF-5z (1, 2, or 4 μM) 1 h before LPS (1 μg/ml) stimulation for 24 h (NO and PGE_2_). The supernatant was obtained and nitrite production in culture media was determined using Griess reaction which is presumed to reflect NO levels. PGE_2_ levels in cell culture media were quantified by PGE_2_ enzyme immunoassay (EIA) kits (Enzo Life Sciences, Inc., NY, United States). After incubation with various doses of TMS-HDMF-5z for 24 h, cells were treated with MTT solution (5 mg/ml) in each well for 4 h at 37°C. MTT formazan was dissolved by adding dimethyl sulfoxide (DMSO), and the absorbance of each well was read by a microplate reader at 540 nm (Molecular Devices, Sunnyvale, CA, United States).

### Protein Extraction and Western Blotting

RAW 264.7 macrophages were seeded at 5 × 10^5^/ml/well in 60 mm^2^ dishes and incubated with TMS-HDMF-5z (1, 2, or 4 μM) in the cultured macrophages with or without the LPS (1 μg/ml). The total protein was lysed in protein extraction solution PRO-PREP (Intron Biotechnology, Seoul, Republic of Korea) and protein concentration was determined using the Bio-Rad protein assay reagent (Bio-Rad Laboratories Inc., CA, United States) according to the manufacturer’s instruction. Protein samples were loaded onto 10% SDS-PAGE gel for electrophoresis separation and were transferred to PVDF membranes. The membrane was incubated overnight with 1:1000 dilution of primary antibody at 4°C. The membrane was washed three times with Tween 20/Tris-buffered saline (T/TBS) and added with a 1:2000 dilution of HRP-conjugated secondary antibody (Santa Cruz Biotechnology Inc., CA, United States) for 2 h at room temperature. The membrane was washed three times with T/TBS and then captured by Western Blotting Luminol Reagent (Santa Cruz Biotechnology, Inc., CA, United States). The relative optical density ratio was determined using a densitometric analysis program (Bio-Rad Quantity One Software, ver 4.6.3 (Basic, Bio-Rad Laboratories Inc., CA, United States) and normalized to the internal control.

### RNA Extraction and Quantitative Real-Time RT-PCR

Total RNA was extracted from the RAW 264.7 macrophages or paw tissues of carrageenan-induced rats using Easy Blue®kit (Intron Biotechnology, Seoul, Republic of Korea). Extracted RNA was reversely transcribed using TOPscript™ RT Dry MIX (Enzynomics, Daejeon, Republic of Korea). Quantitative real-time PCR was performed using a Thermal Cycler Dice Real-Time PCR system using SYBR Premix Ex Taq (Takara Bio Inc., Shiga, Japan). The relative mRNA expression of iNOS, COX-2, TNF-α, IL-1β, IL-6, IFN-β, Ly6C, and F4/80 were adjusted by *ß*-actin expression. The PCR primers used in this study are listed in Table 1.

**TABLE 1:** The list of primer sequence for qRT-PCR.

Specie	Target Gene	Forward	Reverse
Mouse	iNOS	CAT​GCT​ACT​GGA​GGT​GGG​TG	CAT​TGA​TCT​CCG​TGA​CAG​CCC
	COX-2	GGA​GAG​ACT​ATC​AAG​ATA​GT	ATG​GTC​AGT​AGA​CTT​TTA​CA
	TNF-α	AGC​ACA​GAA​AGC​ATG​ATC​CG	CTG​ATG​AGA​GGG​AG-GCC​ATT
	IL-1β	TGC​AGA​GTT​CCC​CAA​CTG​GTA​CAT​C	GTG​CTG​CCT​AAT​GTC​CCC​TTG​AAT​C
	IL-6	GAG​GAT​ACC​ACT​CCC​AAC​AGA​CC	AAG​TGC​ATC​ATC​GTT​GTT​CAT​ACA
	IFN-β	GCA​GCT​GAA​TGG​AAA​GAT​CA	TCC​AGG​AGA​CGT​ACA​ACA​AT
	β-actin	ATC​ACT​ATT​GGC​AAC​GAG​CG	TCA​GCA​ATG​CCT​GGG​TAC​AT
Rat	LY6C	ATG​AAC​AGT​TCT​TGC​GCT​AT	ACC​TGA​GAA​ACA​CAC​ACT​CC
	F4/80	TAC​AGA​GAC​GGG​GTT​TAT​CT	GAT​GAA​AAT​CTG​GGC​AAT​GG
	GAPDH	GTT​ACC​AGG​GCT​GCC​TTC​TC	TGA​CCA​GCT​TCC​CAT​TCT​CA

### Plasmid, Transient Transfection, and Promoter Activity

The transfection with pGL3-iNOS, pGL3-COX-2, pNF-κB-Luc, and pAP-1-Luc and luciferase assay was carried out as described previously (Kim et al., 2019). After 6 h of transfection, cells were pretreated with TMS-HDMF-5z (4 μM) for 15, 30, or 60 min and then stimulated with or without LPS (1 μg/ml) for 18 h. Each well was washed with cold-PBS and cells were lysed and the luciferase activity was determined using the luciferase assay system (Promega Corp., WI, United States).

### Nuclear Extraction and Electrophoretic Mobility Shift Assay

RAW 264.7 macrophages were seeded at 5 × 10^5^/ml/well in 100 mm^2^ dishes and pretreated with TMS-HDMF-5z (4 μM), and then stimulated with LPS (1 μg/ml) for 15, 30, or 60 min. Nuclear extraction and the analysis of binding activity to biotin-labeled NF-κB or AP-1 oligonucleotides were conducted as described previously (Kim et al., 2019).

### Animals

All experiments in the present study were conducted under the university guideline of the ethical committee for Animal Care and Use of the Kyung Hee University according to an animal protocol (KHUASP(SE)-19-185). Sprague-Dawley (SD) male rats (5 weeks) were obtained from the Orient Bio Inc. (Seongnam-si, Republic of Korea) and maintained under constant conditions (temperature: 20 ± 5°C, humidity: 40–60%, light/dark cycle: 12 h).

### Induction of Carrageenan-Induced Hind Paw Edema

SD rats were randomly divided into four groups (*n* = 6): 1) carrageenan-induced group, 2) carrageenan + celecoxib (20 mg/kg, per oral (p.o)), 3) carrageenan + TMS-HDMF-5z (5 mg/kg, intraperitoneal (i.p.)), 4) carrageenan + TMS-HDMF-5z (25 mg/kg, i.p.). Celecoxib and TMS-HDMF-5z were first dissolved in the vehicle solution (DMSO: kolliphor EL:sterile saline = 1:1:18), and then rats were administered intraperitoneal in doses of 5 and 25 mg/kg. After 1 h, a 1% solution of carrageenan in saline (0.1 ml per rat) was injected subcutaneously into the right hind paws. Paw volumes were measured at 1, 3, and 5 h after injections using a water plethysmometer (Panlab, Barcelona, Spain). The increase in paw volume was calculated by subtracting the initial paw volume (basal) from the paw volume measured at each time point. Rats were sacrificed at the end of the experiment, and the paw tissues and blood were collected from each rat for further experiments. To measure ALT, AST, and BUN levels, serum samples were examined using kits from T&P BIO (Gwangju, Republic of Korea).

### Induction of Croton Oil-Induced Ear Edema

The groups are divided equally with the carrageenan-induced paw edema group. SD male rats were pretreated with TMS-HDMF-5z (5 or 25 mg/kg, i.p.) or celecoxib (20 mg/kg, p.o.). After 1 h, ear edema was induced on the inner surface of the left ear by topical application of croton oil (5% solution in 100 μL acetone). The right ear was used as a control and received the same amount of the vehicle (acetone). After 2 h, the ear volume of rats was analyzed using a caliper.

### Statistical Analysis

The results are expressed as the mean ± SD of *in vitro* triplicate and *in vivo* (*n* = 6) experiments. Statistically significant values were compared using ANOVA and Dunnett’s post hoc test, and *p*-values of less than 0.05 were considered statistically significant.

## Results

### TMS-HDMF-5z Inhibits LPS-Induced NO, PGE_2_, and Pro-inflammatory Cytokines Production Through Suppressing iNOS, COX-2, and Pro-inflammatory Cytokines Expression in RAW 264.7 Macrophages

To confirm the reported anti-inflammatory potential of TMS-HDMF-5z (Hassan et al., 2019), its cytotoxic effects were first assessed (1, 2, or 4 μM) on LPS-stimulated RAW 264.7 cells using an MTT assay. As shown in Figure 1B, the cytotoxicity of TMS-HDMF-5z contributing to its suppression on LPS-induced inflammatory mediators is negligible. Next, different concentrations of TMS-HDMF-5z (1, 2, or 4 μM) were pretreated to examine its inhibitory effects on increased NO and PGE_2_ levels caused by LPS treatment. Compared to LPS-treated macrophages, TMS-HDMF-5z exhibited significant suppression of NO and PGE_2_ levels, with the IC_50_ values of 3.95 and 1.26 μM, respectively. L-NIL and NS398 were used as positive controls for NO (62.8 ± 0.81% inhibition at 40 μM) and PGE_2_ production (60.8 ± 3.8% inhibition at 10 nM), respectively. To evaluate whether corresponding gene expression was involved in the inhibition of NO and PGE_2_ by TMS-HDMF-5z treatment, we analyzed the protein and mRNA levels and promoter activity of iNOS and COX-2 enzymes synthesizing NO and PGE_2_. These markers were significantly attenuated in a concentration-dependent manner by the pretreatment of TMS-HDMF-5z (Figures 1C–E). Moreover, the LPS-induced mRNA expression of pro-inflammatory cytokines, such as TNF-α, IL-1β, IL-6, and IFN-β, was significantly downregulated by TMS-HDMF-5z. In particular, the inhibition rates in IL-1β, IL-6, and IFN-β mRNA levels in macrophages activated by LPS were 72, 63, and 55%, respectively, upon treatment with 4 μM TMS-HDMF-5z (Figure 1F).

### TMS-HDMF-5z Inhibits NF-κB Activation and Phosphorylation of IκBα, IKKα/β, and TAK in LPS-Induced RAW 264.7 Macrophages

**FIGURE 2:**
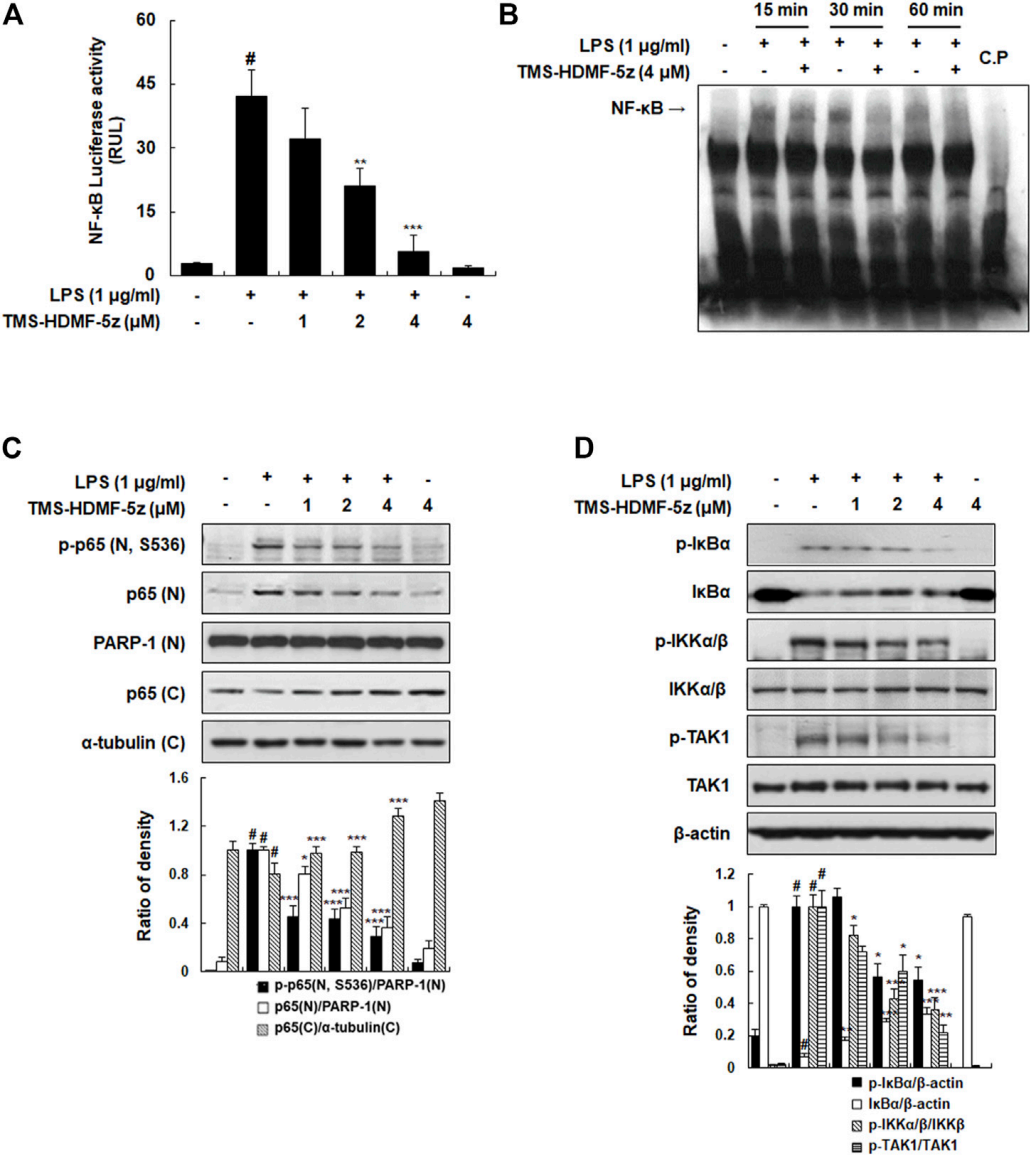
Inhibitory effects of TMS-HDMF-5z on LPS-stimulated NF-κB activity and phosphorylation of IκBα, IKK, and TAK1 in RAW 264.7 macrophages. **(A)** NF-κB luciferase activity in whole-cell extracts was estimated using the Promega Luciferase Assay System (Promega Corp., WI, United States). **(B)** The DNA binding activity of NF-κB was determined using EMSA. **(C)** The nuclear and cytosolic p-p65 (S536) and p65 proteins were detected using western blotting. The relative optical density ratio *vs*. Poly (ADP-ribose) polymerase-1 (PARP-1) or α-tubulin was determined using a densitometric analysis program (Bio-Rad Quantity One Software, version 4.6.3 (Basic), Bio-Rad Laboratories Inc., CA, United States), normalized to the internal control. **(D)** The protein expression of p-IκBα, IκBα, p-IKKα/β, IKKβ, p-TAK1, and TAK1 was detected by western blotting. The relative optical density ratio *vs*. *ß*-actin or total form was determined using a densitometric analysis program (Bio-Rad Quantity One Software, version 4.6.3 (Basic), Bio-Rad Laboratories Inc., CA, United States), normalized to the internal control. Data are shown as the mean ± S.D. of three independent experiments. ^*#*^ *p* < 0.05 *vs*. the control group, *p < 0.05, **p < 0.01, ***p < 0.001 *vs*. LPS-treated cells.

To determine the molecular mechanism of the TMS-HDMF-5z on the suppression of LPS-induced inflammatory responses, we first investigated whether it blocks the activation of NF-κB which is one of the predominant transcription factors regulating numerous inflammatory mediators including iNOS and COX-2 (Wan and Lenardo, 2010). As shown in Figures 2A,B, TMS-HDMF-5z decreased LPS-induced NF-κB luciferase activity in a concentration-dependent manner and also reduced DNA binding activity of NF-κB in LPS-stimulated RAW 264.7 cells at 15 and 30 min, as determined by EMSA. Usually, NF-κB acts as a transcription factor after nuclear localization, which induces the transcription of multiple genes related to inflammation (Wan and Lenardo, 2010). Accordingly, whether TMS-HDMF-5z prevented the translocation of the p65 NF-κB subunit from the cytosol to the nucleus was investigated. While LPS treatment significantly stimulated the translocation of p-p65 (S536) and p65 to the nucleus, and TMS-HDMF-5z concentration-dependently blocked the phosphorylation and its subsequent nuclear translocation of p65 NF-κB subunit (Figure 2C). It is established that NF-κB activation depends on the phosphorylation of IκBα which is subsequently degraded, resulting in the dissociation of NF-κB and its nuclear translocation. Therefore, the effect of TMS-HDMF-5z on IκBα degradation in LPS-induced RAW 264.7 macrophages was determined. TMS-HDMF-5z was found to inhibit LPS-induced IκBα phosphorylation and degradation (Figure 2D). Known as an upstream regulator of IκBα, protein kinases like IKKα/β and TAK1 are known to affect phosphorylation of IκBα during LPS-induced NF-κB signaling (Wan and Lenardo, 2010). According, we examined whether TMS-HDMF-5z inhibits IKKα/β and TAK1 activation in LPS-induced RAW 264.7 macrophages. Results showed that TMS-HDMF-5z markedly inhibited LPS-induced IKKα/β and TAK1 phosphorylation in a concentration-dependent manner but did not affect the levels of total IKKβ and TAK1 (Figure 2D). These results suggest that TMS-HDMF-5z inhibits the NF-κB activity by impeding TAK1-IKKα/β-IκBα-mediated signaling in response to LPS.

### TMS-HDMF-5z Suppresses AP-1 Activation and Its Components (c-Fos, c-Jun, and ATF2) Phosphorylation in LPS-Induced RAW 264.7 Macrophages

**FIGURE 3:**
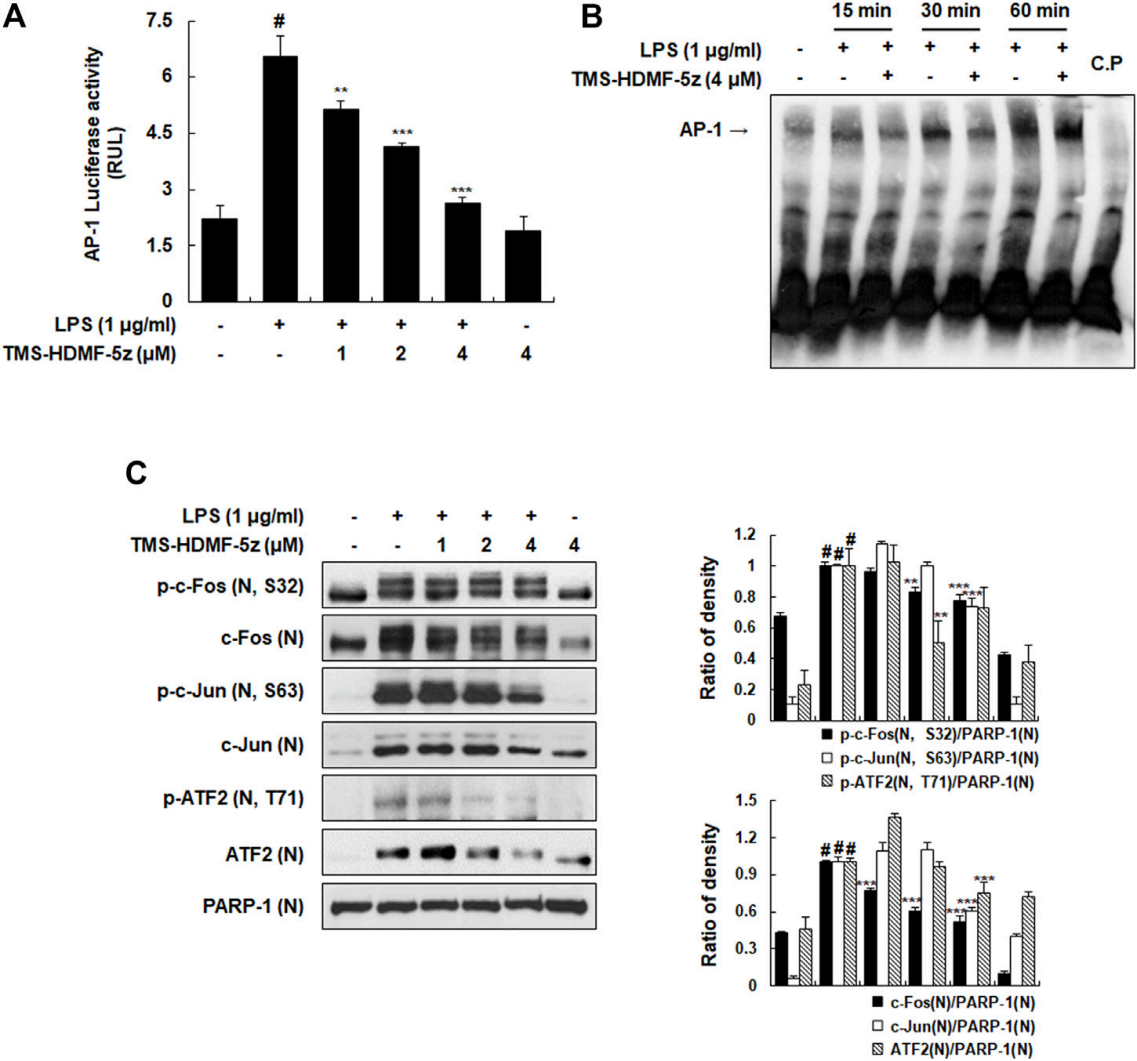
Inhibitory effects of TMS-HDMF-5z on the LPS-induced AP-1 activity and signaling in RAW 264.7 macrophages. **(A)** AP-1 luciferase activity in whole cells extracts was determined using the Promega Luciferase Assay System (Promega Corp., WI, United States). **(B)** The DNA-binding activity of AP-1 was determined using EMSA. **(C)** The nuclear p-c-Fos (S32), c-Fos, p-c-Jun (S63), c-Jun, p-ATF2 (T71), and ATF2 were detected using western blotting. The relative optical density ratio *vs.* PARP-1 was determined using a densitometric analysis program (Bio-Rad Quantity One Software, version 4.6.3 (Basic), Bio-Rad Laboratories Inc., CA, United States), normalized to the internal control. Data are shown as the mean ± S.D. of three independent experiments. ^*#*^ *p* < 0.05 *vs*. the control group, **p < 0.01, ***p < 0.001 *vs*. LPS-treated cells.

Along with NF-κB, the AP-1 complex is also known as another key transcription factor targeting promoter elements of numerous genes coding pro-inflammatory mediators (Cargnello and Roux, 2011). Therefore, a possible inhibition by TMS-HDMF-5z for the activation of the basal AP-1 transcription machinery which is known to be upregulated during inflammation induced by LPS in RAW 264.7 macrophages was explored. TMS-HDMF-5z significantly and concentration-dependently reduced the LPS-stimulated AP-1-dependent luciferase activity (Figure 3A). Moreover, the DNA-binding activity of AP-1 was markedly increased by LPS, and TMS-HDMF-5z (4 μM) attenuated this LPS-induced DNA-binding of AP-1 at 15 and 30 min (Figure 3B). Next, we investigated whether TMS-HDMF-5z inhibited the activation of AP-1 subunits including c-Fos and c-Jun. As shown in Figure 3C, TMS-HDMF-5z decreased the LPS-induced phosphorylation and expression of nuclear c-Fos and c-Jun, suggesting that downregulation of c-Fos and c-Jun by TMS-HDMF-5z can inhibit the formation of AP-1 complex. Since activating transcription factor-2 (ATF2) has been reported to play a role in AP-1 activity by forming homodimers and heterodimers with other members of the ATF family and Jun/Fos family (Cargnello and Roux, 2011), we examined the effects of TMS-HDMF-5z on nuclear ATF2 phosphorylation. TMS-HDMF-5z potently inhibited the LPS-induced nuclear ATF2 phosphorylation and expression in LPS-induced RAW 264.7 macrophages (Figure 3C). Taken together, these results suggest that the inhibitory effects of TMS-HDMF-5z on AP-1 activity induced by LPS were caused by the suppression of nuclear c-Fos, c-Jun, and ATF2 phosphorylation through the reduced expression of these subunits.

### TMS-HDMF-5z Suppresses JAK/STAT Activation in LPS-Induced RAW 264.7 Macrophages

**FIGURE 4:**
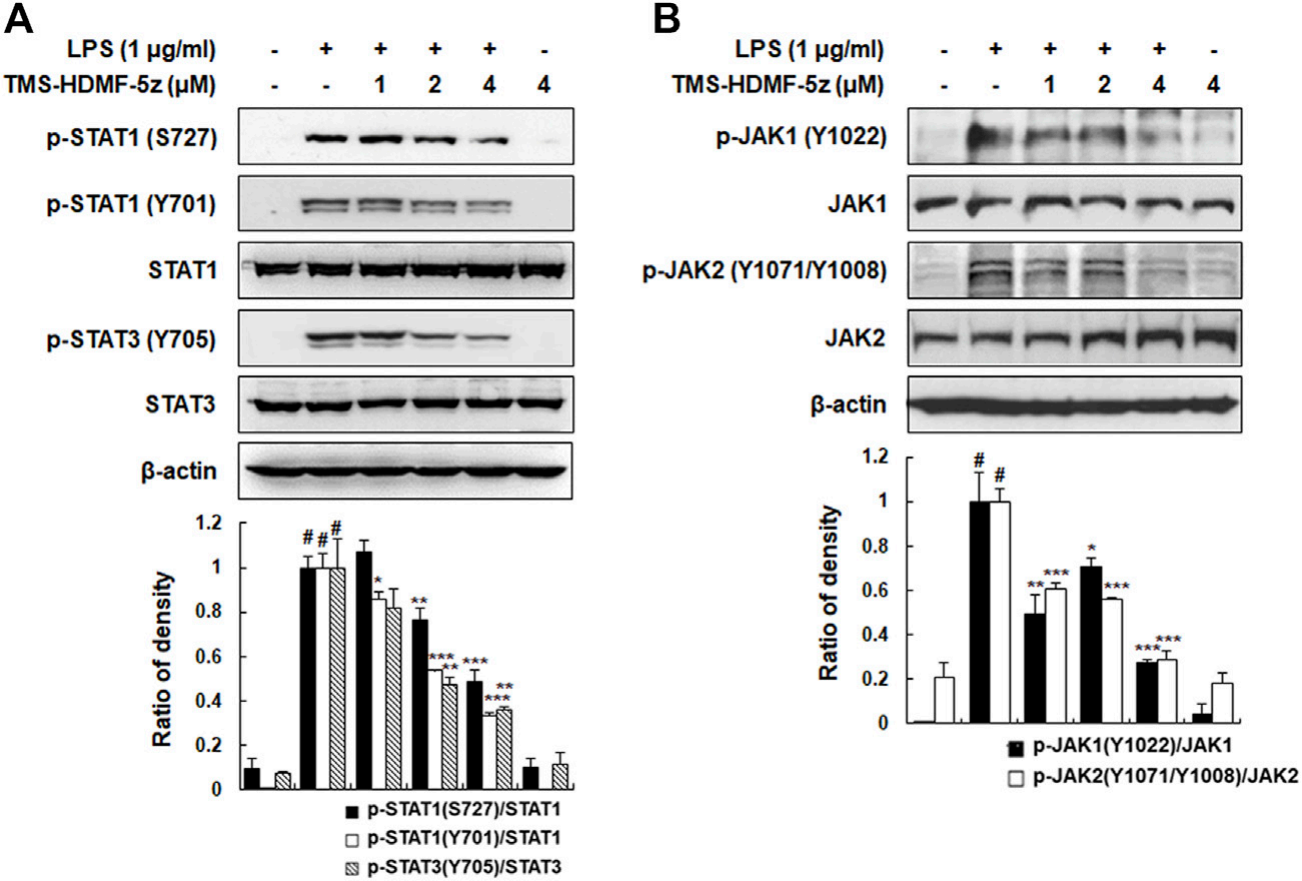
Inhibitory effects of TMS-HDMF-5z on JAK-STAT phosphorylation in LPS-stimulated RAW 264.7 macrophages. **(A,B)** The expression of p-STAT1 (S727, Y701), STAT1, p-STAT3 (Y705), STAT3, p-JAK1 (Y1022), JAK1, p-JAK2 (Y1071/Y1008), and JAK2 was detected by western blotting. The relative optical density ratio *vs*. total form was determined using a densitometric analysis program (Bio-Rad Quantity One Software, version 4.6.3 (Basic), Bio-Rad Laboratories Inc., CA, United States), normalized to the internal control. Data are shown as the mean ± S.D. of three independent experiments. ^*#*^ *p* < 0.05 *vs*. the control group, *p < 0.05, **p < 0.01, ***p < 0.001 *vs*. LPS-treated cells.

The JAK-STAT signaling pathway is the signaling target of several cytokines, LPS, or growth factors, which play important roles in inflammation and thus could also be involved in various diseases, such as skin conditions, cancers, and disorders affecting the immune system (O'Shea and Plenge, 2012). To interrogate whether inhibition of JAK-STAT pathway is involved in mediating the anti-inflammatory effects of TMS-HDMF-5z, the protein levels of p-JAK1, p-JAK2, p-STAT1, and p-STAT3 were measured in LPS-stimulated RAW 264.7 cells. As shown in Figure 4A, TMS-HDMF-5z concentration-dependently inhibited LPS-stimulated phosphorylation of STAT1 (S727 and Y701), which are regulated by p38 MAPK and JAK1, respectively. Besides, we found that LPS-induced JAK2-related STAT3 (Y705) phosphorylation was significantly decreased upon pretreatment with TMS-HDMF-5z. We postulate that TMS-HDMF-5z might be involved in reducing LPS-induced STAT1 (S727) and STAT1/3 (Y701, Y705) levels through p38 MAPK and JAK1/JAK2 inactivation, respectively. LPS exposure markedly induced the phosphorylation of both JAK1 (Y1022) and JAK2 (Y1071/Y1008), and TMS-HDMF-5z significantly attenuated the activation of these proteins to basal levels with no effect on their total protein levels (Figure 4B). These results indicated that TMS-HDMF-5z suppresses the activation of JAK1/JAK2 and p38 MAPK, which subsequently inhibits tyrosine phosphorylation (Y701, Y705) of STAT1/STAT3 and serine phosphorylation (S727) of STAT1 in LPS-induced macrophages, respectively.

### TMS-HDMF-5z Acts as a p38 MAPK Inhibitor in LPS-Induced RAW 264.7 Macrophages

**FIGURE 5:**
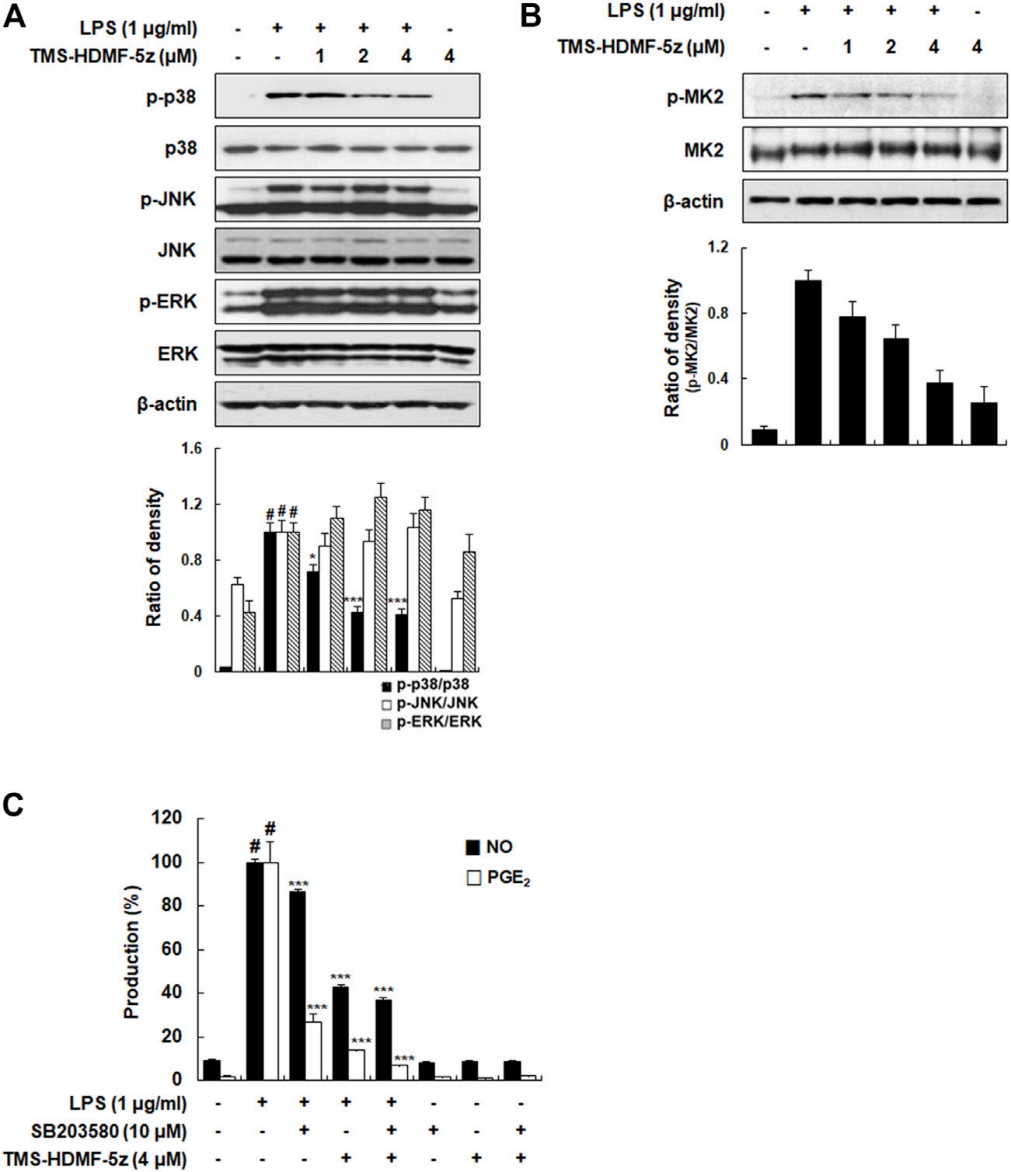
Inhibitory effects of TMS-HDMF-5z on p38 MAPK signaling pathway in LPS-stimulated RAW 264.7 macrophages. **(A,B)** The expression of p-p38 MAPK, p38 MAPK, p-JNK, JNK, p-ERK, ERK, p-MK2, and MK2 were detected by western blotting. The relative optical density ratio *vs*. *ß*-actin was determined using a densitometric analysis program (Bio-Rad Quantity One Software, version 4.6.3 (Basic), Bio-Rad Laboratories Inc., CA, United States), normalized to the internal control. **(C)** NO and PGE_2_ production was determined using the Griess reaction and an EIA kit (Enzo Life Sciences, Inc., NY, United States), respectively. Data are shown as means ± S.D. of three independent experiments. ^*#*^ *p* < 0.05 *vs*. control group, *p < 0.05, **p < 0.01, ***p < 0.001 *vs*. LPS-treated cells.

MAPK plays a vital role in signal transduction pathways in controlling immune responses and inflammatory factors (Kyriakis and Avruch, 2012), and MAPK phosphorylation after LPS stimulation is involved in the activation of transcription factors including AP-1, STAT1, and NF-κB, and subsequently produces inflammatory cytokines (Liu et al., 2018). Thus, the impact of TMS-HDMF-5z treatment on MAPK activation in LPS-induced RAW 264.7 macrophages was investigated. As shown in Figure 5A, TMS-HDMF-5z concentration-dependently inhibited LPS-induced p38 MAPK phosphorylation, while the density of phosphorylated proteins of JNK or ERK1/2 remained unchanged. The levels of total p38 MAPK, JNK, and ERK1/2 were not affected by LPS or TMS-HDMF-5z treatment. These specific inhibitory effects of TMS-HDMF-5z on p38 MAPK activation are consistent with the results of our previous report (Hassan et al., 2019). Since the activation of p38 MAPK leading to the synthesis of pro-inflammatory mediators depends on the phosphorylation of downstream MAPK-activated protein kinase 2 (MK2), ATF2, and STAT1 (Cuadrado and Nebreda, 2010), we further found that TMS-TMF-5z potently decreased the LPS-stimulated phosphorylation of these substrates (Figures 5B, 3C, 4A). To further confirm the involvement of p38 MAPK in inhibiting the production of pro-inflammatory mediators by TMS-HDMF-5z, we examined the effects of SB203580 (10 μM, a specific inhibitor of p38 MAPK) on the production of NO and PGE_2_ with or without TMS-HDMF-5z in LPS-stimulated RAW 264.7 macrophages. As shown in Figure 5C, TMS-HDMF-5z combined with SB203580 more potently inhibited the LPS-induced NO and PGE_2_ production when compared with those of cells treated with only TMS-HDMF-5z or SB203580. These results suggest that the p38 MAPK signaling pathway was specifically involved in the inhibitory effect of TMS-HDMF-5z on LPS-induced NO and PGE_2_ production in RAW 264.7 macrophages.

### TMS-HDMF-5z Attenuates Carrageenan-Induced Paw Edema and Infiltration of Monocytes and Macrophages

**FIGURE 6:**
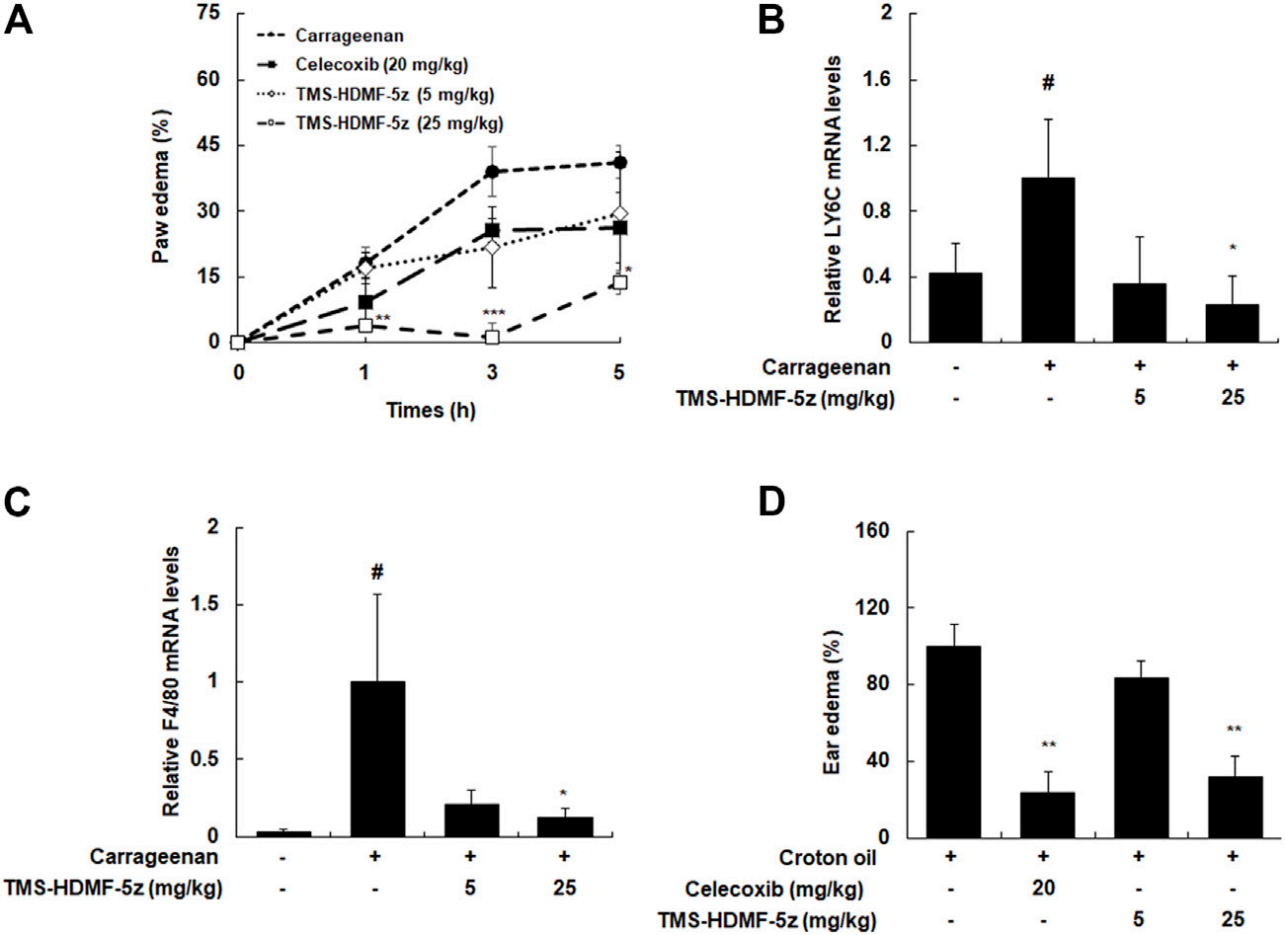
Inhibitory effects of TMS-HDMF-5z administration on carrageenan-induced paw edema and croton oil-stimulated ear edema in rats. **(A)** Paw edema volumes were measured at 1, 3, and 5 h after injection using a plethysmometer. Celecoxib, an anti-inflammatory drug, was used as a positive control. **(B,C)** Total RNAs were prepared from carrageenan-induced paw tissues at 5 h and analyzed for the mRNA expression of Ly6C and F4/80 by qRT-PCR. The levels of Ly6C and F4/80 were normalized to *ß*-actin expression. Data are shown as the mean ± S.D (*n* = 6); ^*#*^ *p* < 0.05 *vs*. control group, *p < 0.05 *vs*. the carrageenan only-treated group. **(D)** Ear edema volumes were measured at 3 h after croton oil application using a caliper. Celecoxib was used as a positive control. Data are shown as the mean ± S.D (*n* = 6). **p < 0.01 *vs*. the croton oil only-treated group.

The *in vivo* anti-inflammatory effects of TMS-HDMF-5z were identified using inflammatory markers, such as paw edema and leukocyte migration, in carrageenan-induced rats. Regarding paw edema, TMS-HDMF-5z (5 or 25 mg/kg, i.p.) treatment inhibited carrageenan-induced paw edema by 43.8 ± 8.1% and 96.8 ± 7.3% at 3 h compared with those of the only carrageenan-induced group, respectively (Figure 6A), while celecoxib (20 mg/kg, p.o.), as a positive control, decreased paw edema by 33.8 ± 4.7% at 3 h. As the infiltration of monocytes and macrophages is important in the pathogenesis of inflammation in carrageenan-induced paw edema (Paterniti et al., 2017), the mRNA expression of Ly6C and F4/80 (well-known biomarkers of monocyte and macrophage, respectively) were examined. As expected, treatment with TMS-HDMF-5z (25 mg/kg, i.p.) potently suppressed the carrageenan-induced mRNA expression of Ly6C and F4/80 (Figures 6B,C). In order to further evaluate the anti-inflammatory activity of TMS-HDMF-5z *in vivo*, croton oil-induced ear edema was studied (Figure 6D). TMS-HDMF-5z (25 mg/kg, i.p.) treatment significantly decreased croton oil-induced ear edema by 68.1 ± 17.6% compared with the group treated with croton oil only, whereas TMS-HDMF-5z (5 mg/kg, i.p.) showed little effects. Celecoxib (20 mg/kg, p.o.) treatment reduced ear edema by 76.7 ± 24.3%.

### TMS-HDMF-5z Ameliorates iNOS and COX-2 Expression Through NF-κB, AP-1, and STAT1/3 Inactivation in Carrageenan-Induced Paw Tissues

**FIGURE 7:**
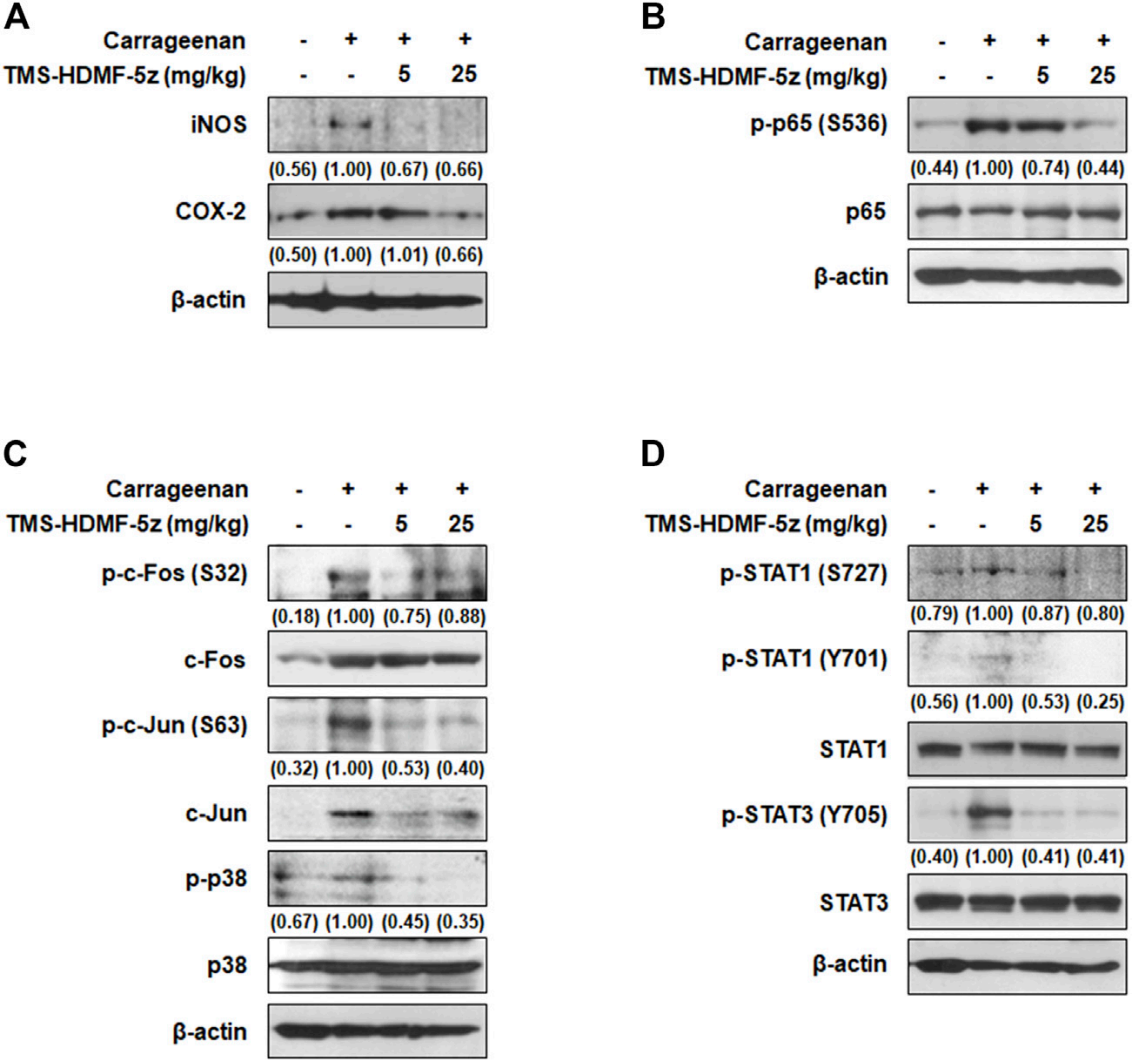
Inhibitory effects of TMS-HDMF-5z on the expression of iNOS, COX-2, and inflammatory transcription factors in carrageenan-induced paw tissues. **(A-D)** Paw tissues were homogenized after 5 h of TMS-HDMF-5z treatment, and whole proteins were prepared for western blotting to detect the protein expression of iNOS, COX-2, p-p65 (S536), p65, p-c-Fos (S32), c-Fos, p-c-Jun (S63), c-Jun, p-p38 MAPK, p38 MAPK, p-STAT1 (S727, Y701), STAT1, p-STAT3 (Y705), and STAT3. The relative optical density ratio *vs.* total form was determined using a densitometric analysis program (Bio-Rad Quantity One Software, version 4.6.3 (Basic), Bio-Rad Laboratories Inc., CA, United States), normalized to the internal control.

In carrageenan-induced paw edema, the expression levels of iNOS and COX-2 are upregulated through the activation of transcription factors (Paterniti et al., 2017). The protein expression of iNOS and COX-2 in paw tissues was upregulated by carrageenan. However, treatment with TMS-HDMF-5z (25 mg/kg, i.p.) potently inhibited this carrageenan-induced upregulation (Figure 7A). Furthermore, we examined the activation of p65, c-Fos, c-Jun, p38, STAT1 (S727, Y701), and STAT3 (Y705) in paw tissues to explore the mechanisms involved in the inhibition of iNOS and COX-2 by TMS-HDMF-5z using western blotting. The phosphorylation of p65, c-Fos, c-Jun, STAT1 (S727, Y701), and STAT3 (Y705) was greatly increased by carrageenan treatment; however, treatment with TMS-HDMF-5z (5 or 25 mg/kg, i.p.) potently attenuated the carrageenan-induced phosphorylation of these proteins (Figures 7B–D). These results suggest that TMS-HDMF-5z relieves carrageenan-induced paw edema by suppressing iNOS and COX-2 expression *via* inactivating p38 MAPK and NF-κB, AP-1, and STAT1/3 transcription factors.

## Discussion

Our previous study demonstrated that TMS-HDMF-5z is a promising new chemical entity that potently inhibits pro-inflammatory cytokines and p38 MAPK phosphorylation in LPS-stimulated RAW 264.7 macrophages (Hassan et al., 2019). However, the detailed molecular mechanisms underlying these anti-inflammatory effects of TMS-HDMF-5z had not yet been fully identified. Thus, in this study, we investigated the signaling pathway underlying the anti-inflammatory properties of TMS-HDMF-5z in LPS-stimulated RAW 264.7 macrophages and the carrageenan-induced paw edema model.

Inflammatory stimuli result in the release of many inflammatory mediators such as NO, PGE_2_, and pro-inflammatory cytokines (TNF-α, IL-1β, IL-6, and IFN-β). These releases are initiated by transcription factor binding to promoter regions and the transcription of respective genes. Therefore, we confirmed our previous results that TMS-HDMF-5z decreased the LPS-induced NO, PGE_2_, and pro-inflammatory cytokine production without non-specific cytotoxicity in RAW 264.7 macrophages. In the present study, the anti-inflammatory effects of TMS-HDMF-5z are supported by its suppression on the LPS-stimulated iNOS and COX-2 expression at protein, mRNA, and promoter binding levels, and TNF-α, IL-1β, IL-6, and IFN-β expression at the mRNA level. To further investigate the mechanisms underlying the suppression of these pro-inflammatory mediators of TMS-HDMF-5z, we focused on various transcription factors such as NF-κB, AP-1, and STAT, which play an important role in modulating inflammatory and immune diseases (O'Shea and Plenge, 2012). In macrophages, LPS enhances NF-κB and AP-1 signaling pathways through TLR4 stimulation. TLR4 activates the phosphorylation of TAK1, and then IKK is phosphorylated to facilitate the degradation of IκB. The phosphorylation and degradation of IκB activate the transcription factor p65/p50, known as the important subunit consisting of the NF-κB complex (Lawrence, 2009). Accordingly, we determined whether TMS-HDMF-5z showed an inhibitory effect on NF-κB activity and downstream signaling in LPS-stimulated RAW 264.7 macrophages. We found that TMS-HDMF-5z inhibited the LPS-induced transcription and DNA-binding activity of NF-κB by suppressing nuclear p65 phosphorylation and reducing the phosphorylation and degradation of IκB by inhibiting IKK and TAK1 phosphorylation. LPS also activates the AP-1 transcription factors such as nuclear proteins c-Fos and c-Jun, which are activated by the MAPK pathway, including ERK, JNK, and p38 MAPK in macrophages (Liu et al., 2018). Thus, we showed that TMS-HDMF-5z inhibited the LPS-stimulated promoter and DNA binding activities of AP-1 through nuclear translocation and phosphorylation of c-Fos and c-Jun suppression in LPS-induced RAW 264.7 macrophages. The JAK-STAT signaling pathway is associated with inflammatory, autoimmune, and neoplastic diseases (O'Shea and Plenge, 2012). LPS induces the phosphorylation of the JAK1 and JAK2, which then upregulates tyrosine phosphorylation of STAT1 and STAT3, respectively (O'Shea and Plenge, 2012). Phosphorylation at the Y701 site leads to the translocation of cytosolic STAT1 dimers into the nucleus, where they bind to specific promoter regions of DNA. STAT1 should also be phosphorylated at S727 by p-p38 MAPK for full transcriptional activity, indicating that p38 MAPK could act as an important factor for STAT1 activation (Luu et al., 2014). Additionally, STAT3 is phosphorylated at Y705, facilitating inflammatory responses through its activation, homodimerization, and nuclear translocation, as are STAT1 dimers (Samavati et al., 2009). Accordingly, we found that TMS-HDMF-5z prevented the phosphorylation of JAK1 (Y1022) and JAK2 (Y1071/Y1008) as well as STAT1 (S727 and Y701) and STAT3 (Y705). Moreover, after exposure to LPS, STAT can be activated through autocrine activation *via* the TRIF-IRF3-IFN-β pathway (Park et al., 2014). We found that TMS-HDMF-5z exhibited suppressive effects on LPS-induced STAT1 phosphorylation (Y701) and mRNA expression of IFN-β expression in RAW 264.7 mac-rophages, suggesting that TMS-HDMF-5z could also regulate the autocrine IFN-β-induced activation of JAK-STAT. We suggest that the inhibition of p38 MAPK phosphorylation and IFN-β expression suppressed serine and tyrosine phosphorylation of STAT1, respectively in LPS-induced RAW 264.7 macrophages. In these regards, we could not rule out the suppression effect of TMS-HDMF-5z on synergistic interaction with NF-κB, AP-1, and STAT1/3. NF-κB-dependent transcription is not only tightly controlled by positive and negative regulatory mechanisms but also closely coordinated with other transcription factors (Oeckinghaus et al., 2011). NF-κB p65 can interact directly with both c-Jun and c-Fos and can stimulate the binding of AP-1 to DNA and its activation through AP-1 sites. Congruently, c-Jun and c-Fos can promote transactivation of p65 through κB sites even in the absence of AP-1 sites (Dorrington and Fraser, 2019). Moreover, NF-κB and STAT are rapidly activated in response to various stimuli including stresses and cytokines. Global chromatin binding surveys revealed that STAT binds at least 3,000 different gene promoters and the number of genes targeted by NF-κB family members is even larger. Importantly, NF-κB and STAT control together the expression of an overlapping groups of target genes during inflammation (Grivennikov and Karin, 2010). Considerng all of these, we suggested that TMS-HDMF-5z would ameliorate the concomitant activation of NF-κB, AP-1, and STAT1/3, involving the cooperation of these transcription factors.

Furthermore, we explored whether TMS-HDMF-5z attenuated the phosphorylation of MAPK, which is crucial to inflammatory cytokines and signaling pathways. TMS-HDMF-5z decreased the LPS-stimulated phosphorylation of p38 MAPK, whereas it did not affect the phosphorylation of ERK or JNK. Accumulating evidence suggests that p38 MAPK is a major player during inflammatory responses, especially in macrophages (Cuadrado and Nebreda, 2010). There are several substrates downstream of p38 MAPK signaling pathways including MK2, ATF2, and p38α MAPK-related/activated protein kinase (PRAK). The stimulation of MK2 is required for pro-inflammatory cytokines such as IL-1, IL-6, TNF-α, and IFN-β production and cell migration in inflammatory conditions, thus MK2 is likely to be a potential drug target for inflammatory diseases (Ehlting et al., 2019). ATF2 is a transcription factor which plays a role in AP-1 activity that regulates the expression of pro-inflammatory mediator genes by forming homodimers and heterodimers with other members of the ATF family and Jun/Fos family, respectively (Watson et al., 2017). In our experiment, TMS-HDMF-5z suppressed the LPS-induced MK2 and ATF2 phosphorylation in RAW 264.7 macrophages. Information from the present study suggests the inhibition of pro-inflammatory mediators including iNOS, COX-2, TNF-α, IL-1β, IL-6, and IFN-β expression of TMS-HDMF-5z might be partly mediated through the suppression of p38 MAPK phosphorylation, leading to decreased phosphorylation of ATF2 and MK2, pos-sibly reducing AP-1 binding on iNOS, COX-2, TNF-α, IL-1β, IL-6, and IFN-β promoters and/or the mRNA stability of pro-inflammatory mediators. In support of the involvement of the p38 MAPK signaling pathway in inhibiting LPS-induced NO and PGE_2_ production, macrophages were treated with TMS-HDMF-5z and SB203580, a specific p38 MAPK inhibitor. Combination of TMS-HDMF-5z and SB203580 showed an additive inhibitory effects on LPS-induced NO and PGE_2_ production, indicating that they can partly inhibit the same pathway.

To verify the *in vivo* relevance of our *in vitro* results regarding the anti-inflammatory effects of TMS-HDMF-5z, we utilized the carrageenan-induced paw and croton oil-induced edema models. Carrageenan-induced hind paw edema is a preliminary screening method with acceptable reproducibility to evaluate the anti-edematous efficacy of new therapeutic agents. The inflammatory edema caused by carrageenan is the result of the interaction of several inflammatory mediators considered to be biphasic. The initial phase (60–90 min) is related to the release of histamine, bradykinin, serotonin, substance P, and prostaglandins (PGs). After this time, the release of PGs and the infiltration of leukocytes reaches a peak around 4–6 h and maintains edema (Patil et al., 2019). These pro-inflammatory mediators, in combination or separately, recruit inflammatory cells (monocytes, macrophages, and neutrophils) and lead to a systemic inflammatory response. Pretreatment with TMS-HDMF-5z (5 or 25 mg/kg, i.p.) provokes noteworthy inhibitory effects on paw swelling development in two phases of the carrageenan test and recruitment of monocytes and macrophages. During inflammatory conditions, there is enhanced release of inflammatory mediators including NO, PGE_2_, and pro-inflammatory cytokines which are mainly triggered by activated peripheral mono-nucleated phagocytes or other immune cells. Consistent with *in vitro* results, expression levels of iNOS and COX-2 significantly decreased with the application of TMS-HDMF-5z through suppressing p38 MAPK as well as transcription factors such as NF-kB, AP-1, and STAT1/3 in the carrageenan-induced paw edema tissues. Intense inflammatory changes, such as the development of ear edema and inflammatory cell infiltration were caused by croton oil. Pretreatment with TMS-HDMF-5z (25 mg/kg, i.p.) significantly reduced the ear thickness caused by croton oil application. Edema is the typical feature of inflammation not only in systemic inflammation but also in local inflammation (Zhang et al., 2011). Therefore, these results suggest that TMS-HDMF-5z has anti-inflammatory potency in different inflammatory disease such as arthritis and sepsis. In addition, analysis of AST, ALT, and BUN serum levels and body weight did not show any changes (data not shown). Further preclinical studies including toxicological, histopathological, and pharmacokinetic aspects as well as pharmacological evidence are needed for the clinical drug development of TMS-HDMF-5z through complying with the guidelines dictated by Good Laboratory Practice.

In conclusion, our data indicated that TMS-HDMF-5z exerts anti-inflammatory properties by suppressing iNOS and COX-2 expression *via* inactivation of AP-1, NF-κB, and JAK/STAT, along with p38 MAPK signaling pathways in LPS-induced RAW 264.7 macrophages and carrageenan-induced paw edema. Thus, our findings suggest that TMS-HDMF-5z should be investigated further as a potential option for treating inflammatory diseases.

## Data Availability

The original contributions presented in the study are included in the article/Supplementary Material, further inquiries can be directed to the corresponding author.

## Abbreviations

AP-1, activator protein-1; COX-2, cyclooxygenase-2; IFN-β, interferon-β; IKK, IκB kinase; IκBα, IκB kinase; IL, interleukin; JAK 1/2, Janus kinase 1/2; MAPK, mitogen-activated protein kinase; MK2, MAPK-activated protein kinase 2; NF-κB, nuclear factor-κB; STAT 1/3, signal transducers and activators of transcription 1/3; TAK1, TGF-β activated kinase 1; TF2, activating transcription factor 2; TNF-α, tumor necrosis factor-α.
