# Supplementary material for: Mosloflavone-Resveratrol Hybrid TMS-HDMF-5z Exhibits Potent In Vitro and In Vivo Anti-Inflammatory Effects Through NF-κB, AP-1, and JAK/STAT Inactivation
Source: Front Pharmacol. 2022 Apr 21;13:857789. doi: 10.3389/fphar.2022.857789 (PMC9068937; doi:10.3389/fphar.2022.857789)
Supplement: Supplementary file 1 [file Presentation1.PPTX]

## Slide 1
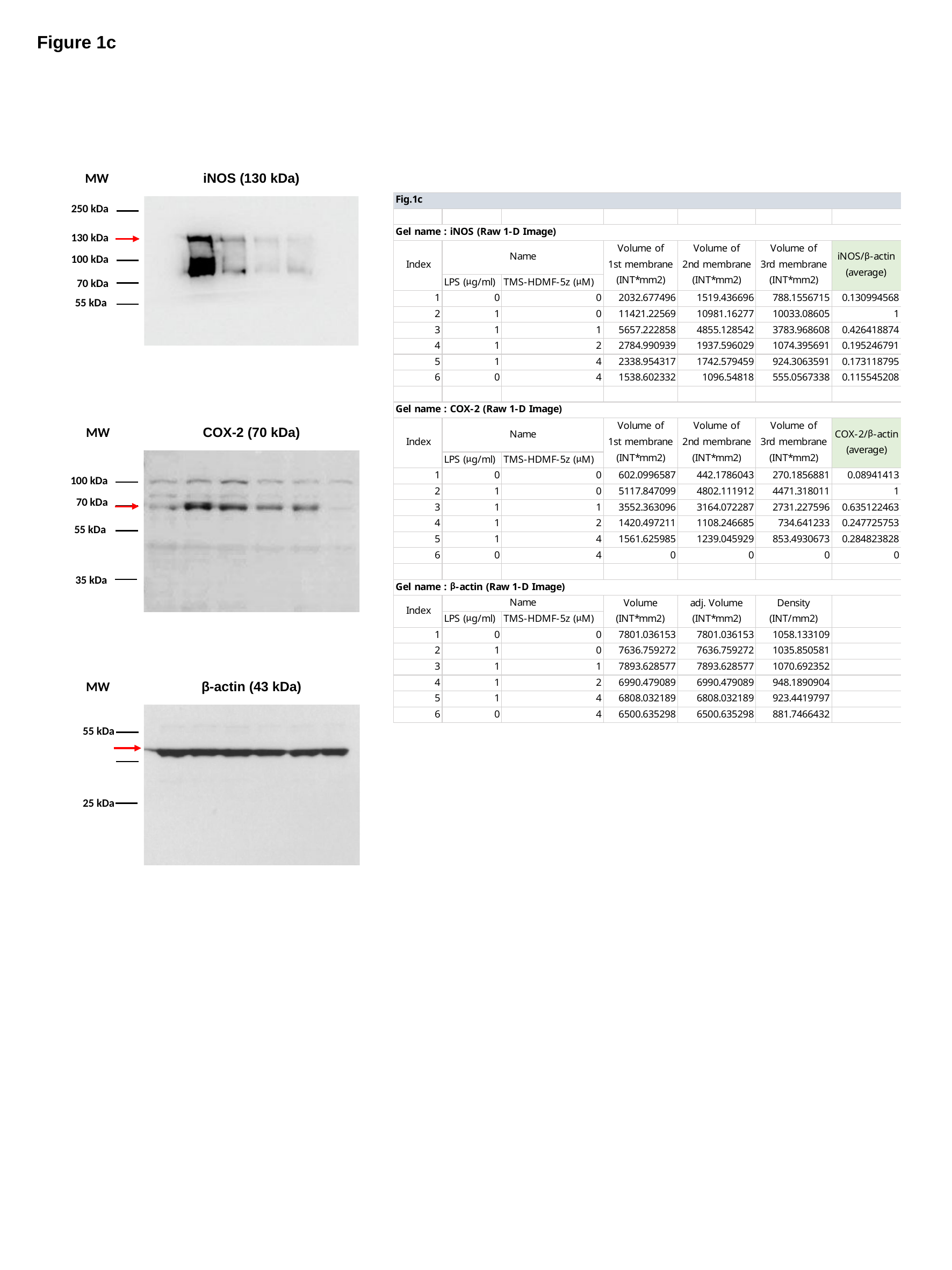

Figure 1c
iNOS (130 kDa)
MW
250 kDa
130 kDa
100 kDa
70 kDa
55 kDa
MW
COX-2 (70 kDa)
100 kDa
70 kDa
55 kDa
35 kDa
MW
β-actin (43 kDa)
55 kDa
25 kDa

## Slide 2
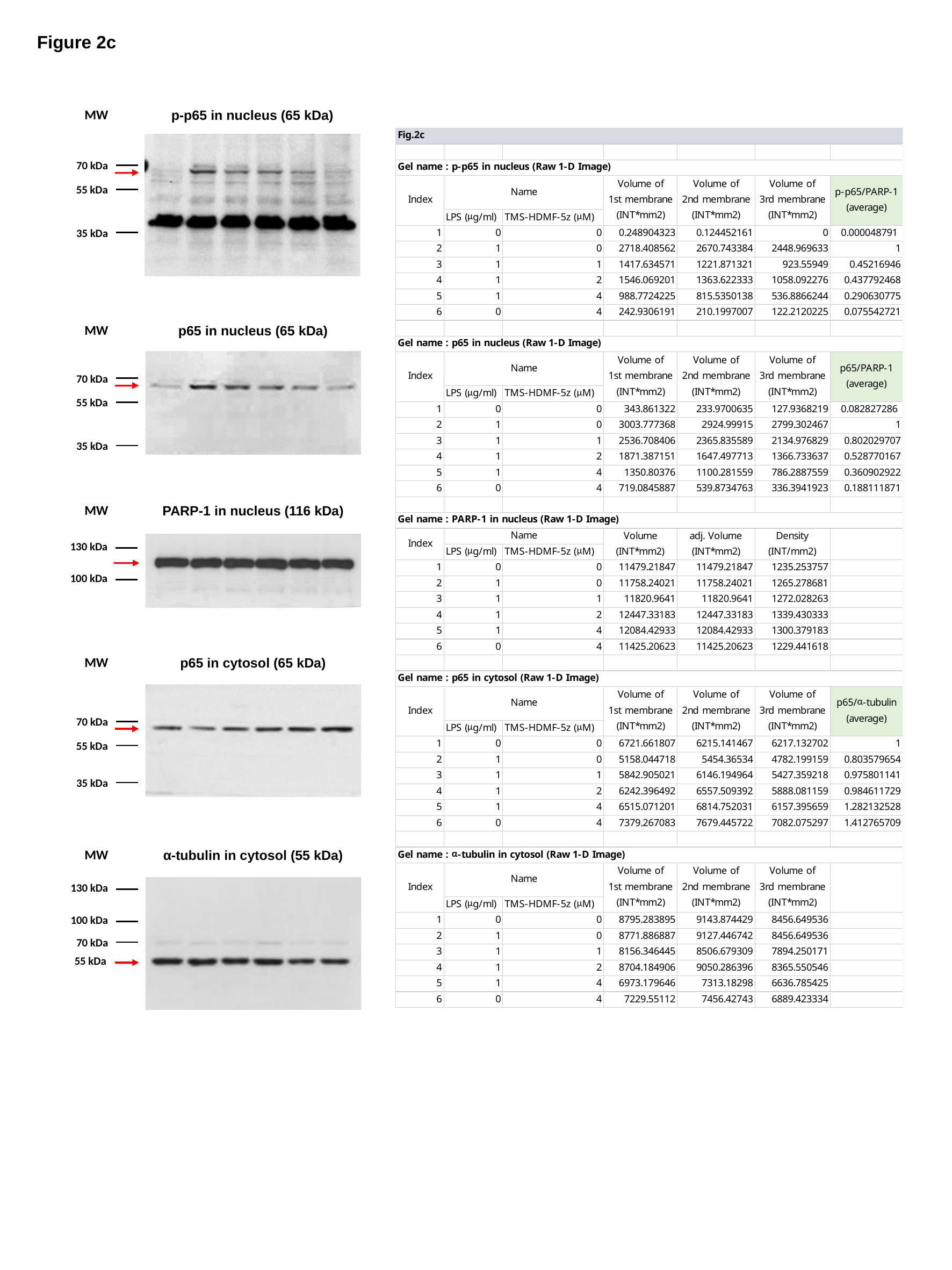

Figure 2c
MW
p-p65 in nucleus (65 kDa)
70 kDa
55 kDa
35 kDa
MW
p65 in nucleus (65 kDa)
70 kDa
55 kDa
35 kDa
MW
PARP-1 in nucleus (116 kDa)
130 kDa
100 kDa
MW
p65 in cytosol (65 kDa)
70 kDa
55 kDa
35 kDa
MW
α-tubulin in cytosol (55 kDa)
130 kDa
100 kDa
70 kDa
55 kDa

## Slide 3
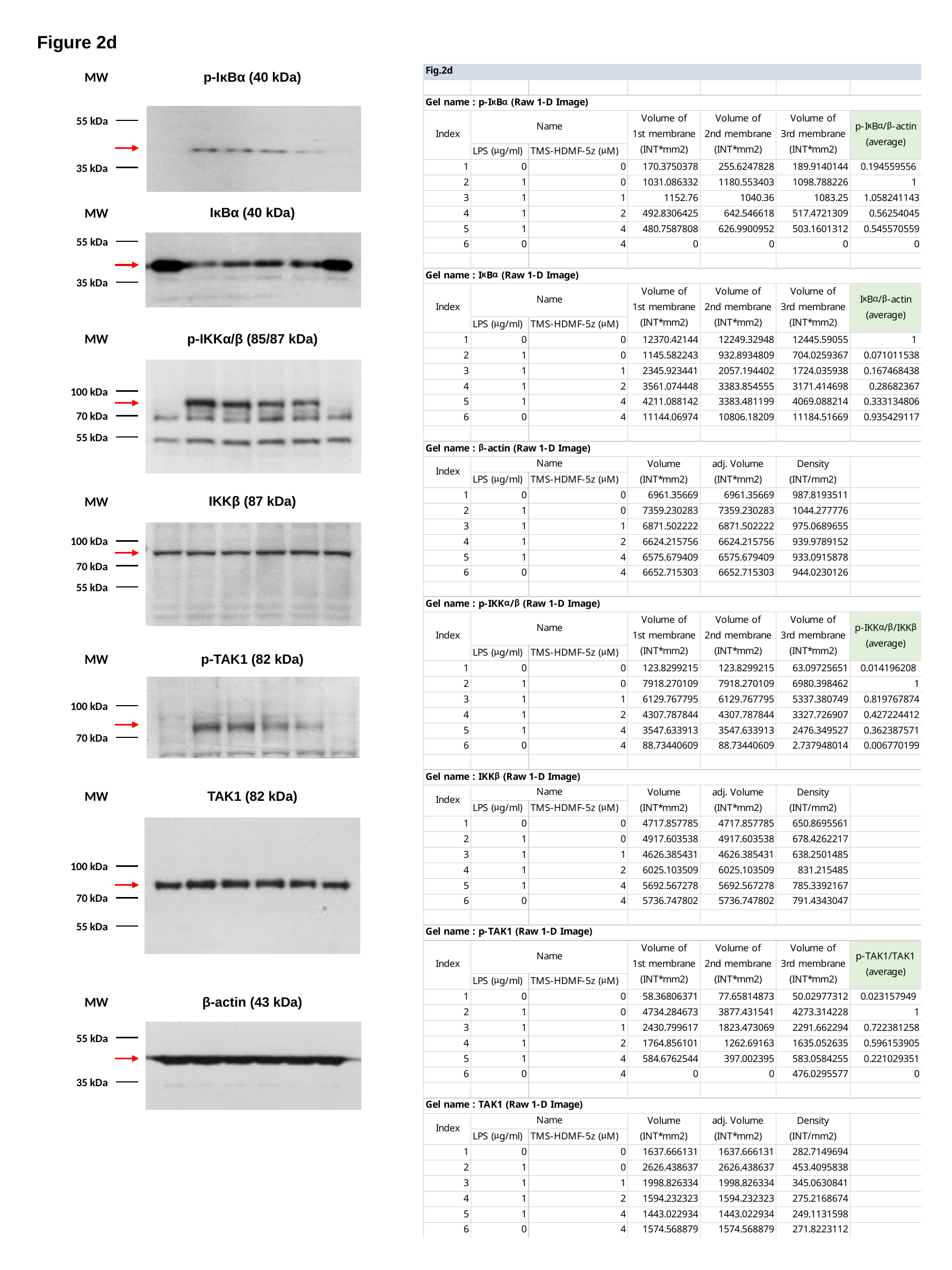

Figure 2d
p-IκBα (40 kDa)
MW
55 kDa
35 kDa
IκBα (40 kDa)
MW
55 kDa
35 kDa
p-IKKα/β (85/87 kDa)
MW
100 kDa
70 kDa
55 kDa
IKKβ (87 kDa)
MW
100 kDa
70 kDa
55 kDa
p-TAK1 (82 kDa)
MW
100 kDa
70 kDa
TAK1 (82 kDa)
MW
100 kDa
70 kDa
55 kDa
β-actin (43 kDa)
MW
55 kDa
35 kDa

## Slide 4
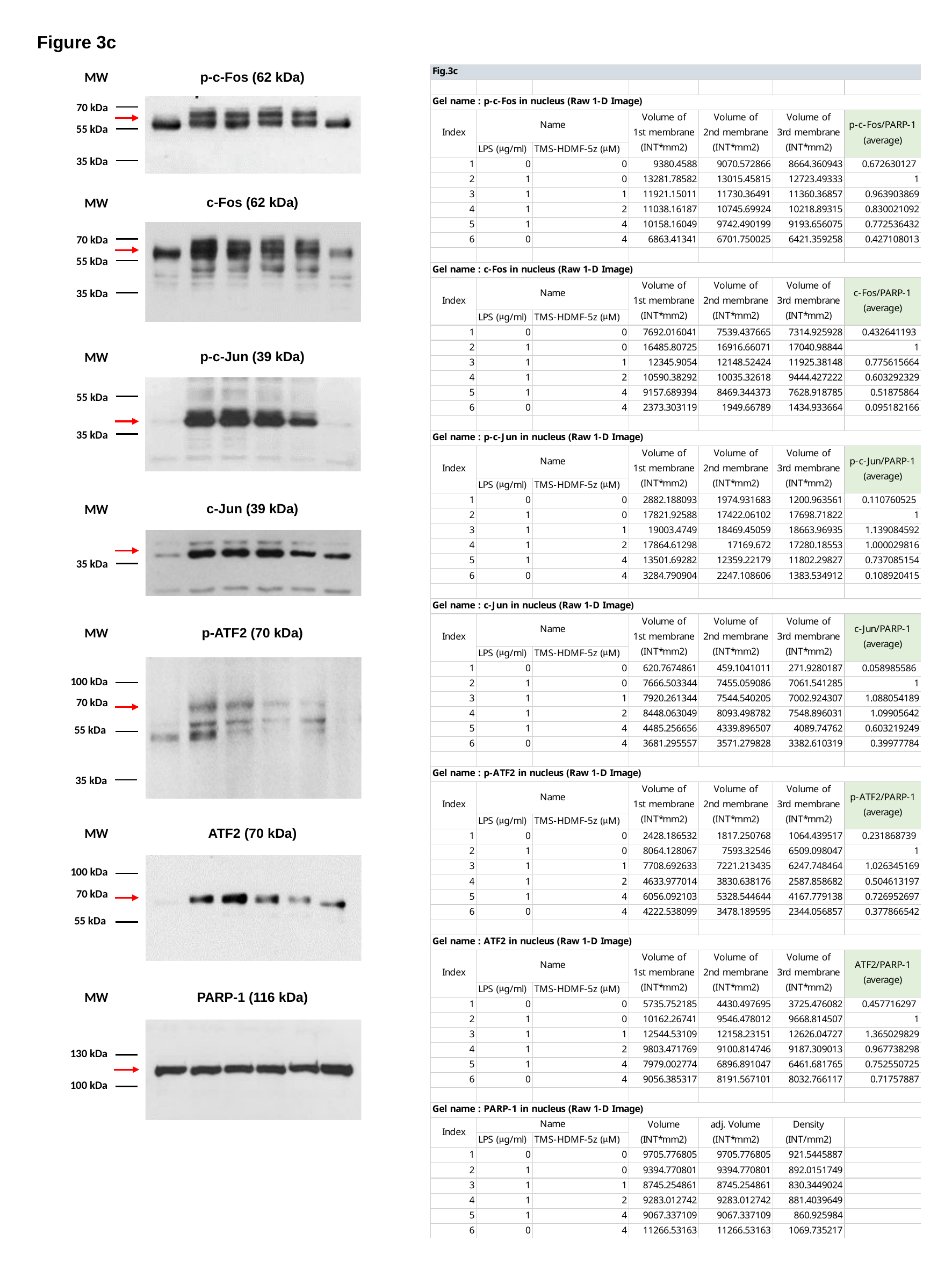

Figure 3c
p-c-Fos (62 kDa)
MW
70 kDa
55 kDa
35 kDa
c-Fos (62 kDa)
MW
70 kDa
55 kDa
35 kDa
p-c-Jun (39 kDa)
MW
55 kDa
35 kDa
c-Jun (39 kDa)
MW
35 kDa
p-ATF2 (70 kDa)
MW
100 kDa
70 kDa
55 kDa
35 kDa
ATF2 (70 kDa)
MW
100 kDa
70 kDa
55 kDa
PARP-1 (116 kDa)
MW
130 kDa
100 kDa

## Slide 5
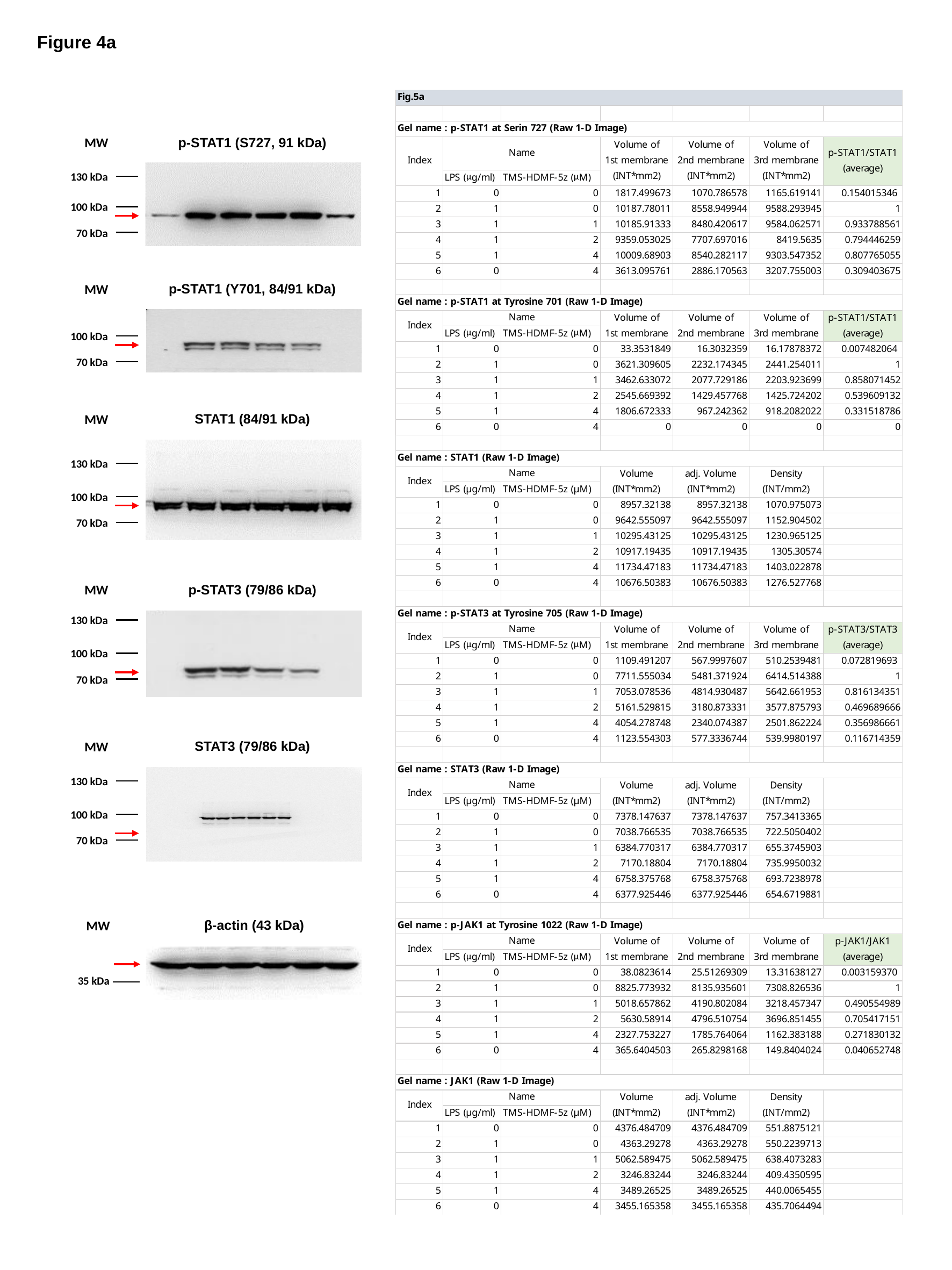

Figure 4a
p-STAT1 (S727, 91 kDa)
MW
130 kDa
100 kDa
70 kDa
p-STAT1 (Y701, 84/91 kDa)
MW
100 kDa
70 kDa
STAT1 (84/91 kDa)
MW
130 kDa
100 kDa
70 kDa
p-STAT3 (79/86 kDa)
MW
130 kDa
100 kDa
70 kDa
STAT3 (79/86 kDa)
MW
130 kDa
100 kDa
70 kDa
β-actin (43 kDa)
MW
35 kDa

## Slide 6
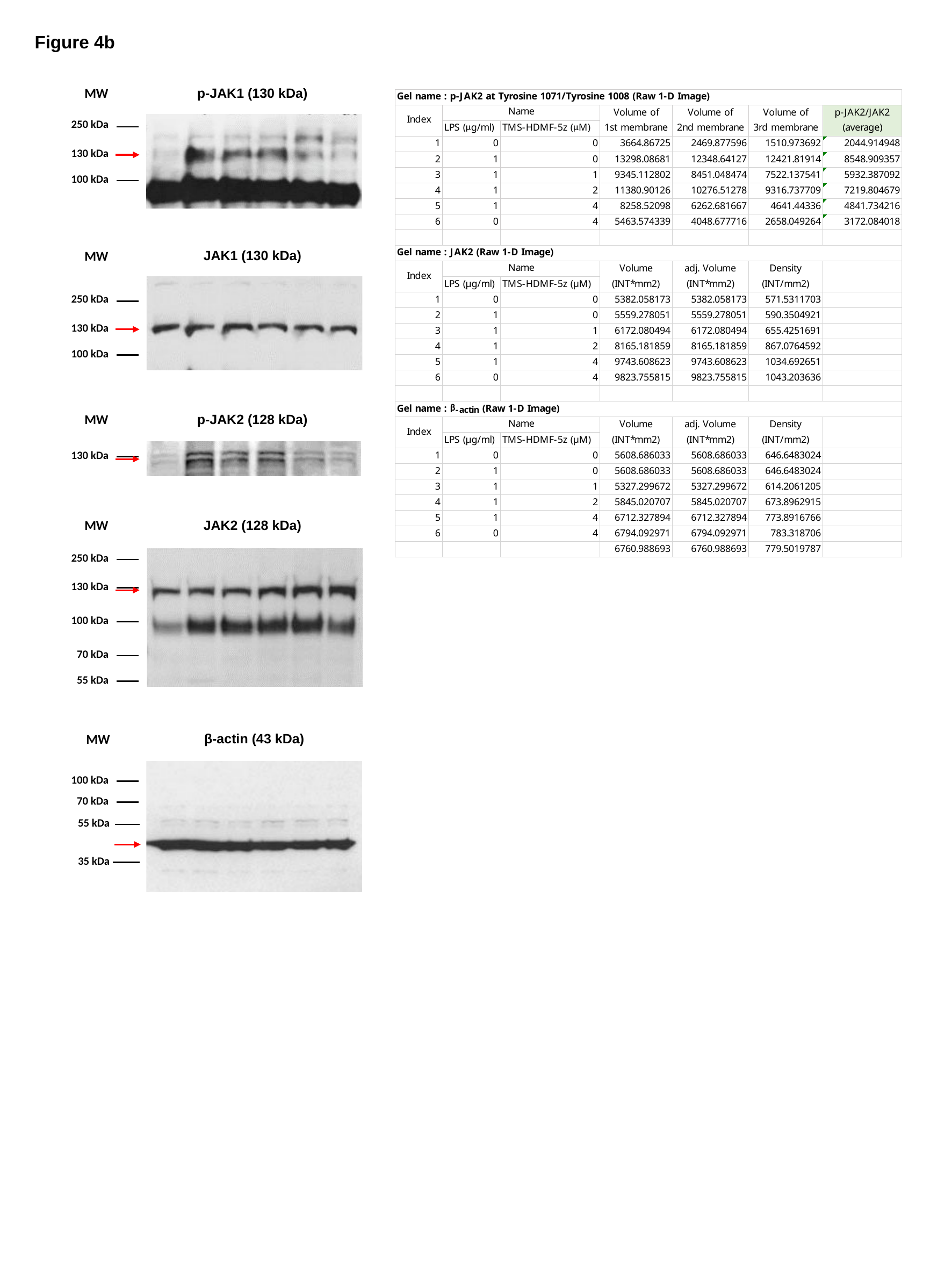

Figure 4b
p-JAK1 (130 kDa)
MW
250 kDa
130 kDa
100 kDa
JAK1 (130 kDa)
MW
250 kDa
130 kDa
100 kDa
p-JAK2 (128 kDa)
MW
130 kDa
JAK2 (128 kDa)
MW
250 kDa
130 kDa
100 kDa
70 kDa
55 kDa
β-actin (43 kDa)
MW
100 kDa
70 kDa
55 kDa
35 kDa

## Slide 7
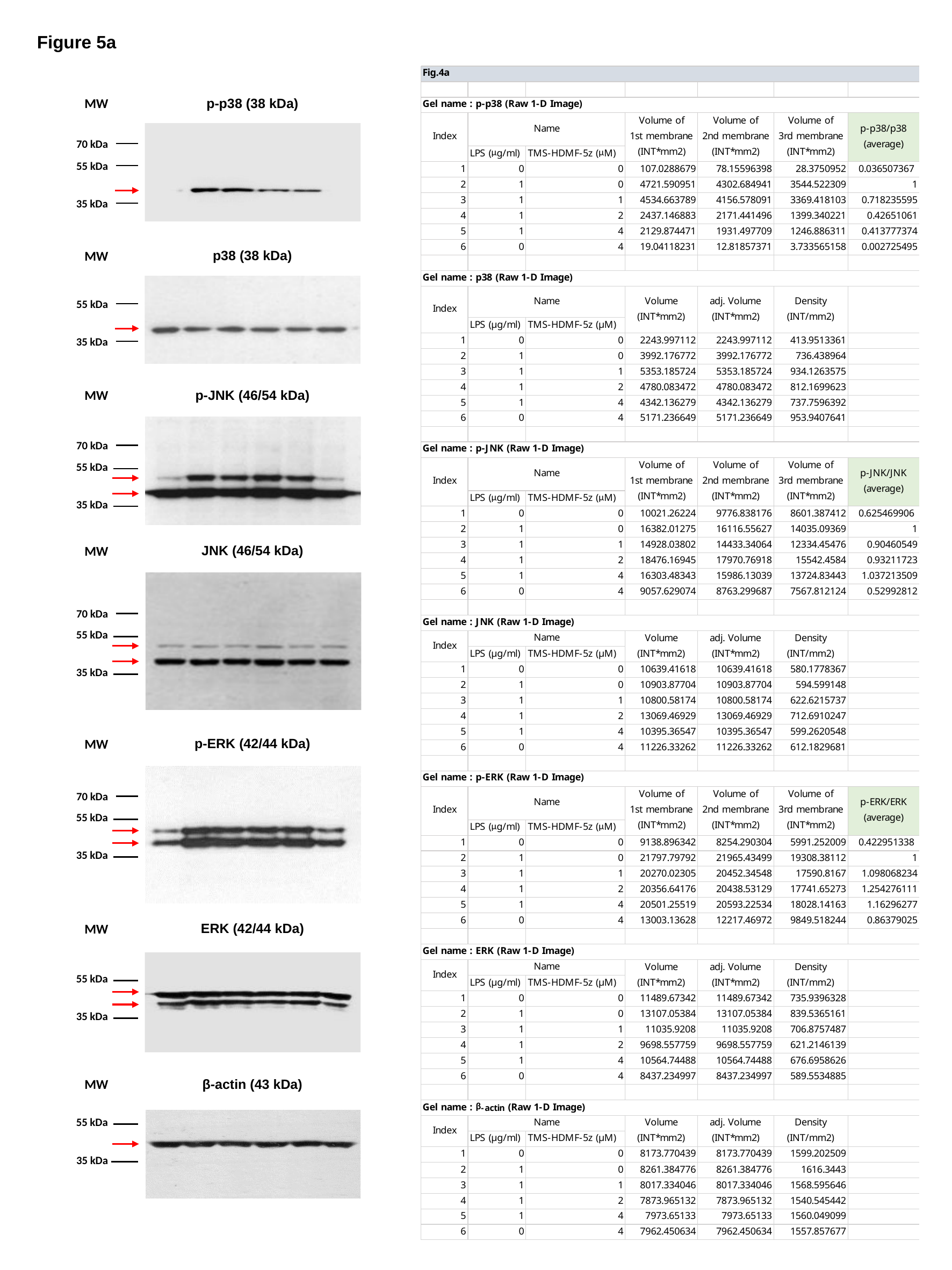

Figure 5a
p-p38 (38 kDa)
MW
70 kDa
55 kDa
35 kDa
p38 (38 kDa)
MW
55 kDa
35 kDa
p-JNK (46/54 kDa)
MW
70 kDa
55 kDa
35 kDa
JNK (46/54 kDa)
MW
70 kDa
55 kDa
35 kDa
p-ERK (42/44 kDa)
MW
70 kDa
55 kDa
35 kDa
ERK (42/44 kDa)
MW
55 kDa
35 kDa
β-actin (43 kDa)
MW
55 kDa
35 kDa

## Slide 8
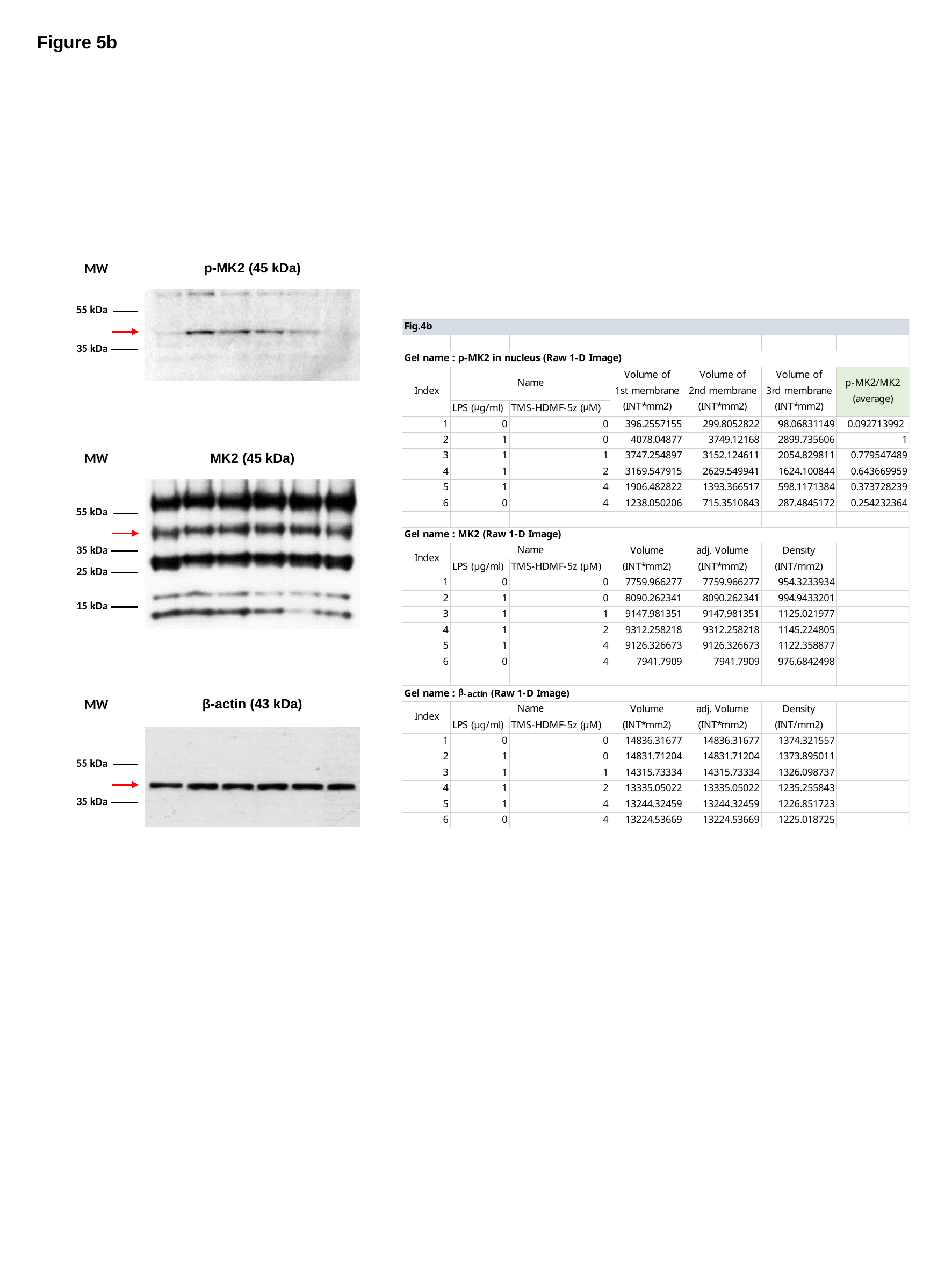

Figure 5b
p-MK2 (45 kDa)
MW
55 kDa
35 kDa
MK2 (45 kDa)
MW
55 kDa
35 kDa
25 kDa
15 kDa
β-actin (43 kDa)
MW
55 kDa
35 kDa

## Slide 9
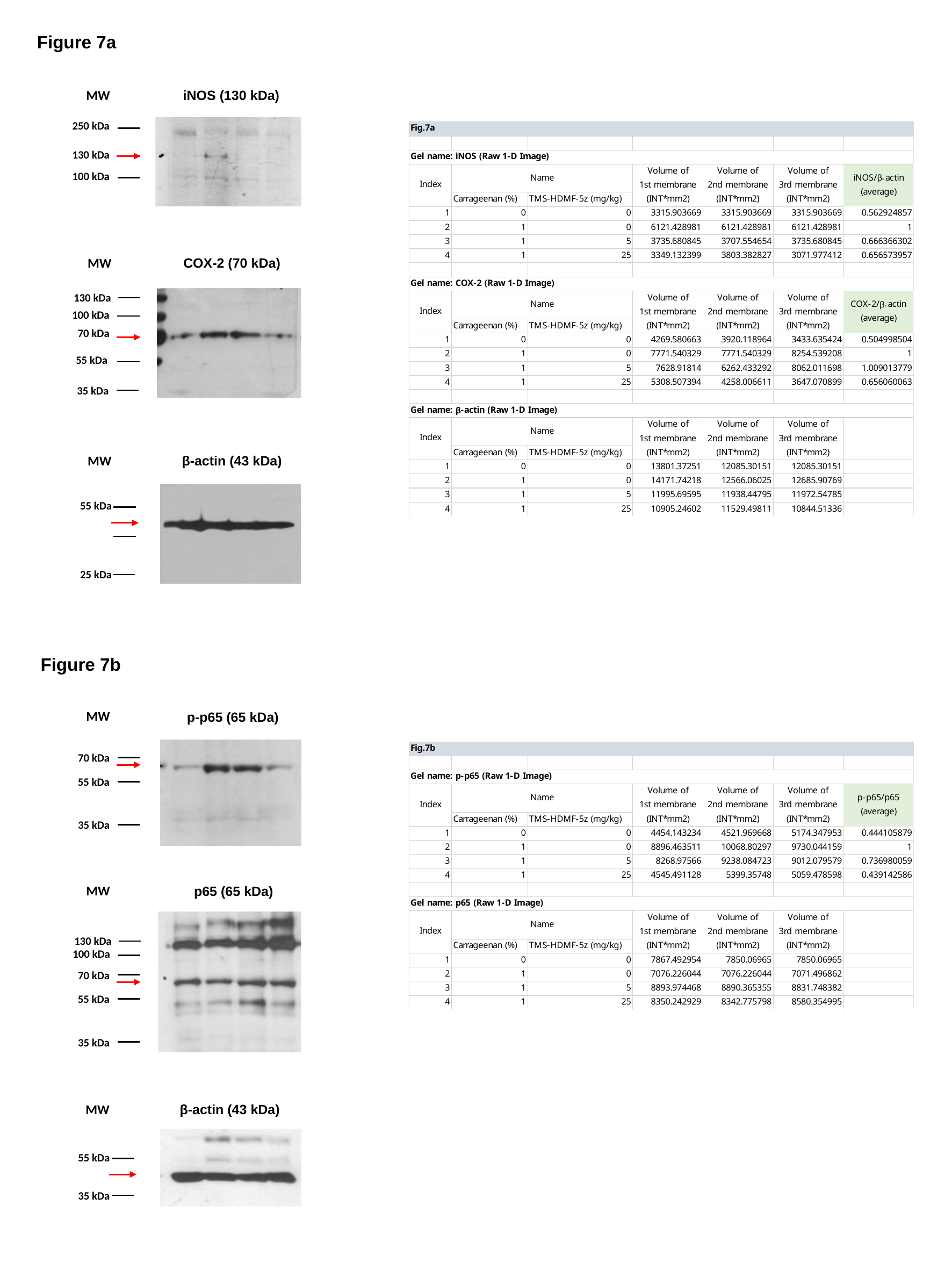

Figure 7a
iNOS (130 kDa)
MW
250 kDa
130 kDa
100 kDa
MW
COX-2 (70 kDa)
130 kDa
100 kDa
70 kDa
55 kDa
35 kDa
MW
β-actin (43 kDa)
55 kDa
25 kDa
Figure 7b
MW
p-p65 (65 kDa)
70 kDa
55 kDa
35 kDa
MW
p65 (65 kDa)
130 kDa
100 kDa
70 kDa
55 kDa
35 kDa
MW
β-actin (43 kDa)
55 kDa
35 kDa

## Slide 10
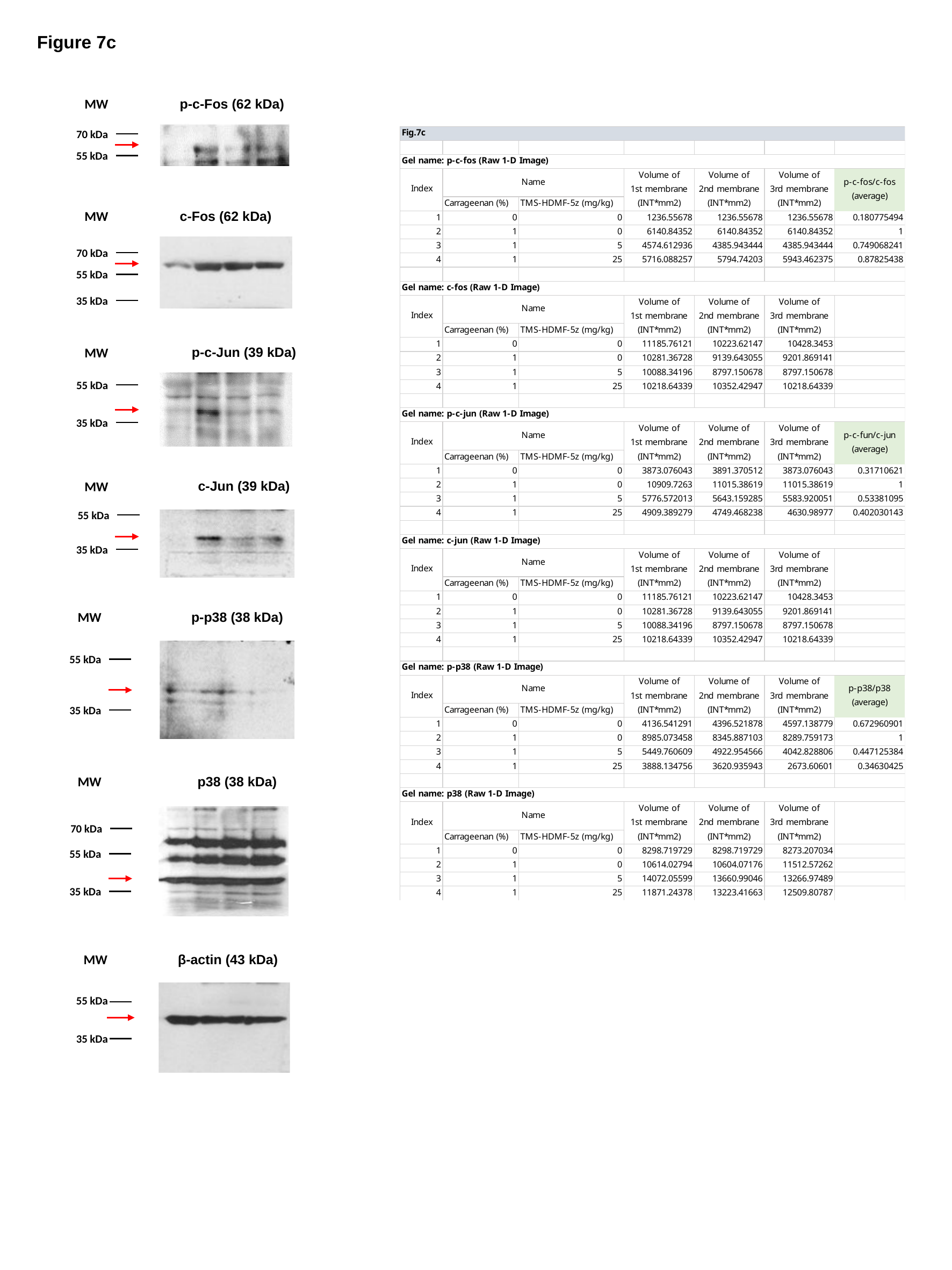

Figure 7c
p-c-Fos (62 kDa)
MW
70 kDa
55 kDa
c-Fos (62 kDa)
MW
70 kDa
55 kDa
35 kDa
p-c-Jun (39 kDa)
MW
55 kDa
35 kDa
c-Jun (39 kDa)
MW
55 kDa
35 kDa
p-p38 (38 kDa)
MW
55 kDa
35 kDa
p38 (38 kDa)
MW
70 kDa
55 kDa
35 kDa
MW
β-actin (43 kDa)
55 kDa
35 kDa

## Slide 11
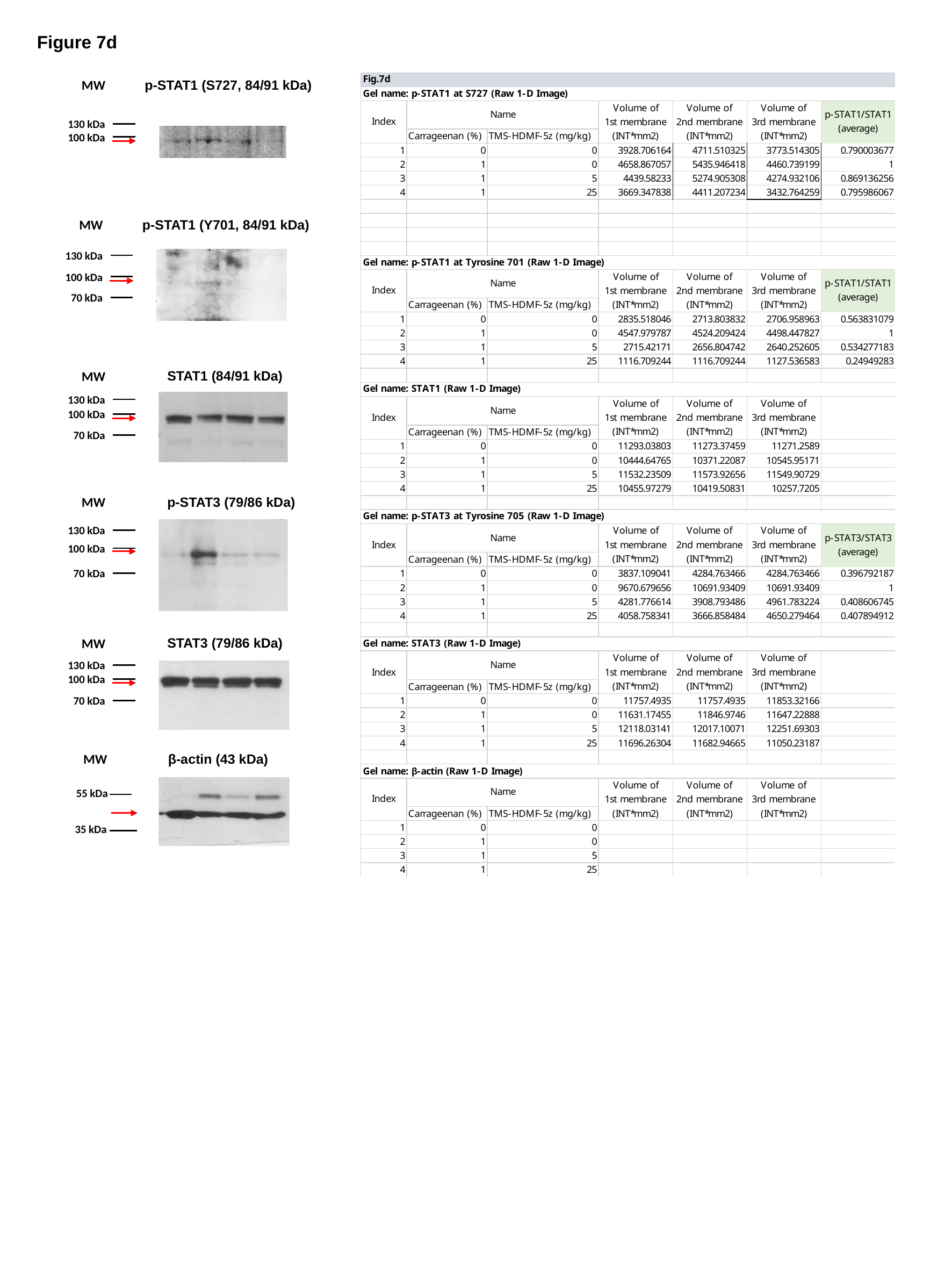

Figure 7d
p-STAT1 (S727, 84/91 kDa)
MW
130 kDa
100 kDa
p-STAT1 (Y701, 84/91 kDa)
MW
130 kDa
100 kDa
70 kDa
STAT1 (84/91 kDa)
MW
130 kDa
100 kDa
70 kDa
p-STAT3 (79/86 kDa)
MW
130 kDa
100 kDa
70 kDa
STAT3 (79/86 kDa)
MW
130 kDa
100 kDa
70 kDa
β-actin (43 kDa)
MW
55 kDa
35 kDa
