# Supplementary figures and images for: Mosloflavone-Resveratrol Hybrid TMS-HDMF-5z Exhibits Potent In Vitro and In Vivo Anti-Inflammatory Effects Through NF-κB, AP-1, and JAK/STAT Inactivation
Source: Front Pharmacol. 2022 Apr 21;13:857789. doi: 10.3389/fphar.2022.857789 (PMC9068937; doi:10.3389/fphar.2022.857789)

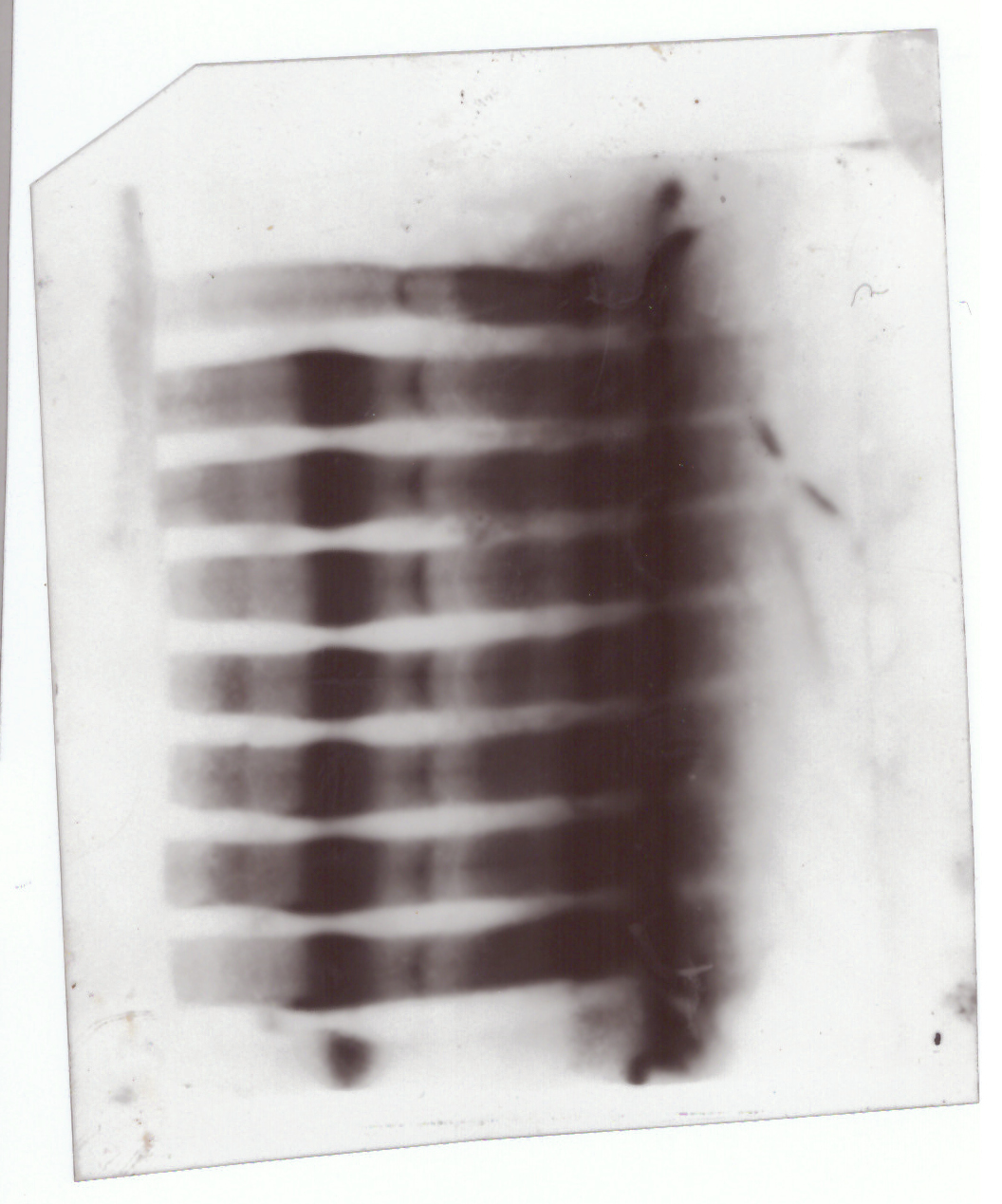

Supplement: Supplementary file 2 [file DataSheet1.ZIP › 4H raw data (SOOK)(KYS)/Figure 2/B/Scan_20180810_NF-KB ╜├░ú║░ 15,30,60min.jpg]

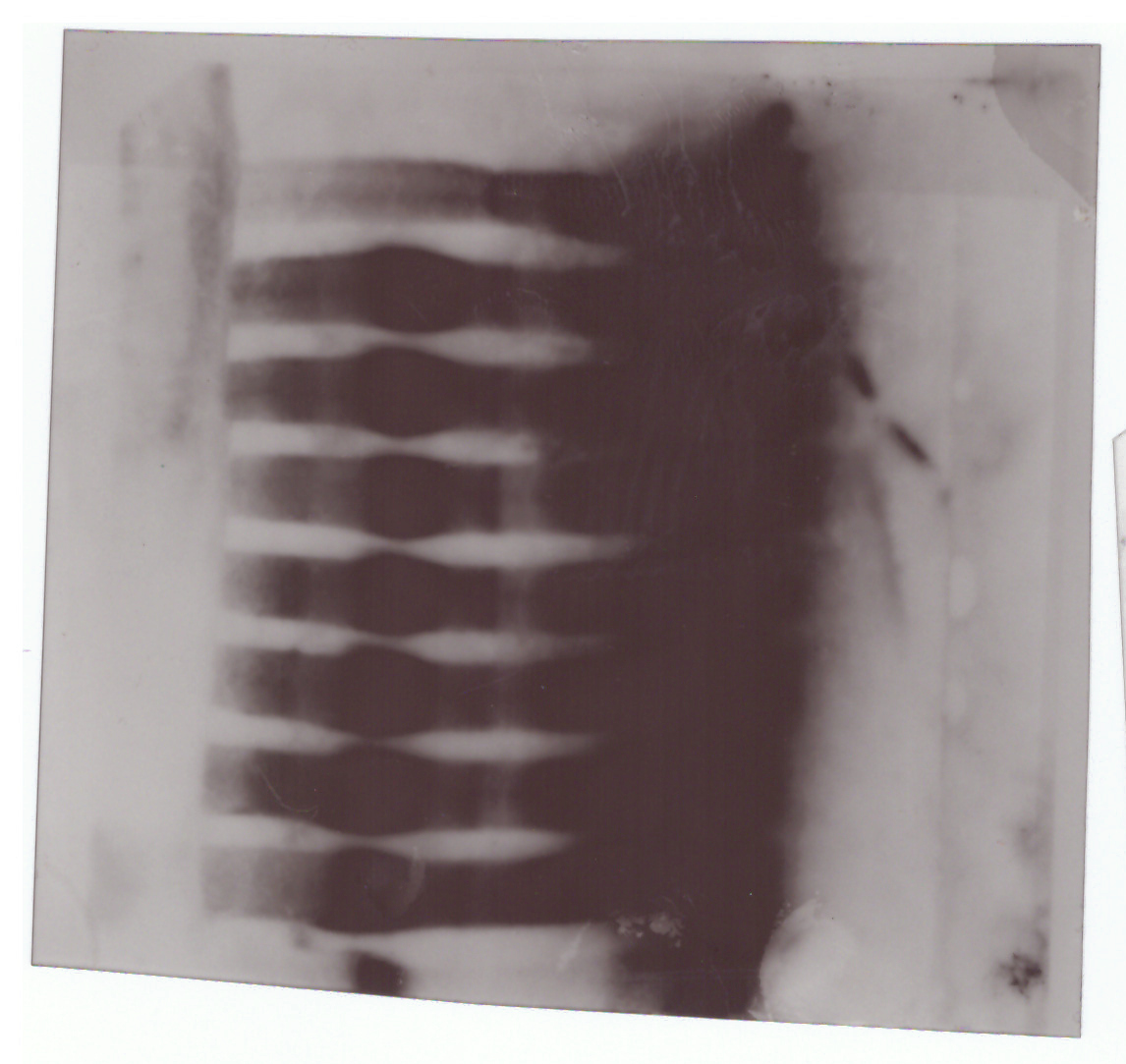

Supplement: Supplementary file 2 [file DataSheet1.ZIP › 4H raw data (SOOK)(KYS)/Figure 2/B/Scan_20180810_NF-KB ╜├░ú║░-1 15,30,60min.jpg]

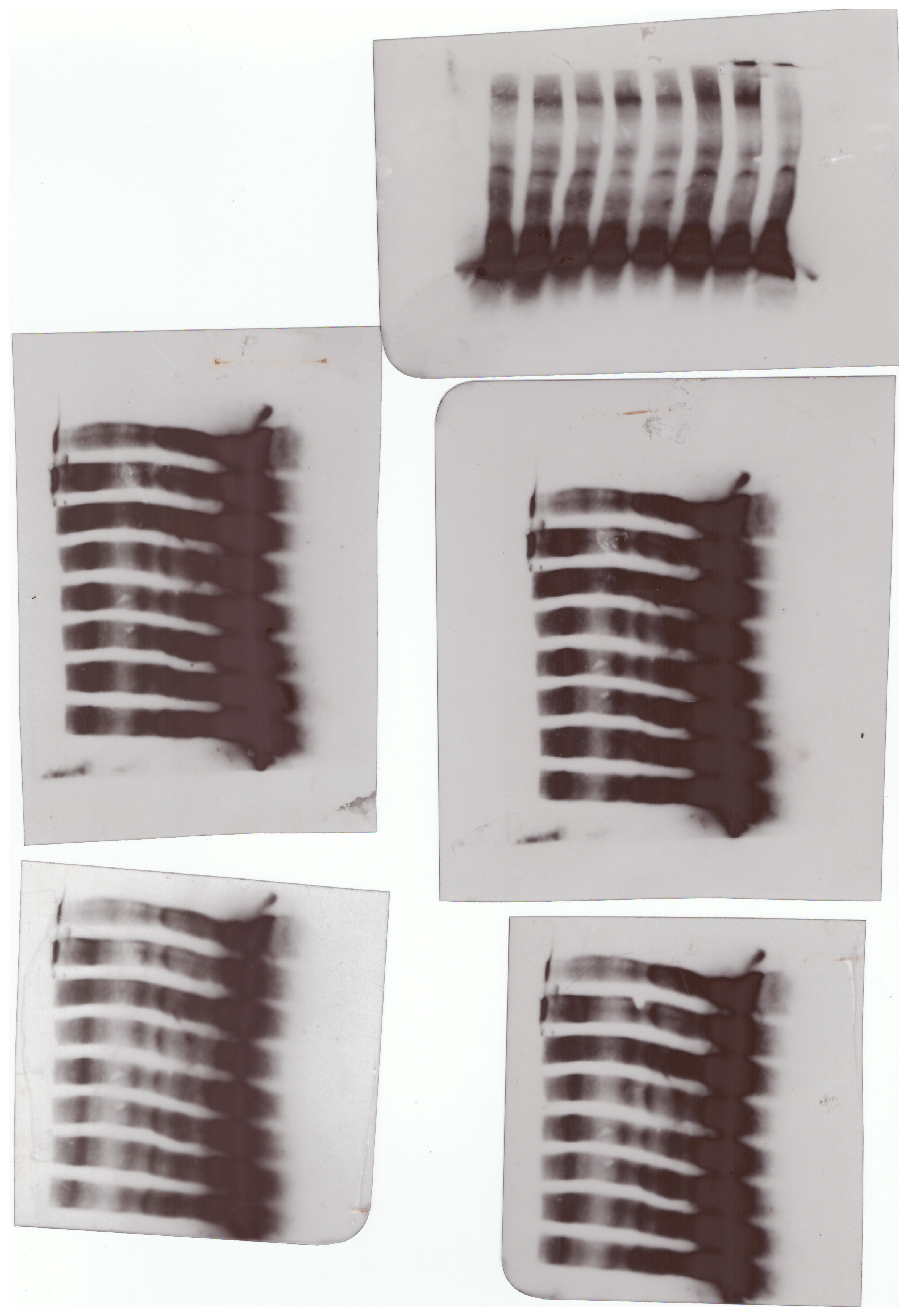

Supplement: Supplementary file 2 [file DataSheet1.ZIP › 4H raw data (SOOK)(KYS)/Figure 3/B/Scan_20180817_ap-1 ╜├░ú║░ 15,30,60min.jpg]
